# Characterization of the gut microbiota and fecal metabolome in the osteosarcoma mouse model

**DOI:** 10.18632/aging.205951

**Published:** 2024-07-03

**Authors:** Yuan Li, Xiaochen Qiao, Yi Feng, Ruhao Zhou, Kun Zhang, Yongchun Pan, Ting Yan, Lei Yan, Sen Yang, Xiaochun Wei, Pengcui Li, Chaojian Xu, Zhi Lv, Zhi Tian

**Affiliations:** 1 Second Clinical Medical College, Shanxi Medical University, Taiyuan 030001, Shanxi, P.R. China; 2 Department of Orthopedics, The Second Hospital of Shanxi Medical University, Shanxi Key Laboratory of Bone and Soft Tissue Injury Repair, Taiyuan 030001, Shanxi, P.R. China; 3 Department of Orthopedics, Third People's Hospital of Datong City, Datong 037006, Shanxi, P.R. China; 4 Translational Medicine Center, Shanxi Medical University, Taiyuan 030001, Shanxi, P.R. China; 5 Department of Orthopedics, The Second People's Hospital of Changzhi, Changzhi 046000, Shanxi, P.R. China

**Keywords:** osteosarcoma, animal model, gut microbiota, 16S rDNA sequencing, non-targeted metabolomics

## Abstract

Previous studies have reported the correlation between gut microbiota (GM), GM-derived metabolites, and various intestinal and extra-intestinal cancers. However, limited studies have investigated the correlation between GM, GM-derived metabolites, and osteosarcoma (OS). This study successfully established a female BALB/c nude mouse model of OS. Mice (n = 14) were divided into the following two groups (n = 7/group): OS group named OG, injected with Saos-2 OS cells; normal control group named NCG, injected with Matrigel. The GM composition and metabolites were characterized using 16S rDNA sequencing and untargeted metabolomics, respectively. Bioinformatics analysis revealed that amino acid metabolism was dysregulated in OS. The abundances of bone metabolism-related genera *Alloprevotella, Rikenellaceae_RC9_gut_group*, and *Muribaculum* were correlated with amino acid metabolism, especially histidine metabolism. These findings suggest the correlation between GM, GM-derived metabolites, and OS pathogenesis. Clinical significance: The currently used standard therapeutic strategies for OS, including surgery, chemotherapy, and radiation, are not efficacious. The findings of this study provided novel insights for developing therapeutic, diagnostic, and prognostic strategies for OS.

## INTRODUCTION

Osteosarcoma (OS), a common primary malignant tumor, accounts for 20% of all primary malignant bone tumors. Children, adolescents, and young adults aged 10–25 years are commonly affected by OS [1], which is characterized by a high degree of malignancy, increased recurrence, and early lung metastasis [2]. Recent advances in therapeutic methods for OS, including neoadjuvant chemotherapy, surgical resection of the primary tumor, and adjuvant chemotherapy [3], have not improved the 5-year survival rates for localized disease (50%–70%) and metastatic and recurrent OS (less than 20%) [4].

The human microbiome comprises various microorganisms (such as bacteria, fungi, archaea, protozoa, and viruses) and inhabits the surface of the human epithelial barrier. The intestinal microbiome is strongly associated with host health and disease status [5–7]. Gut microbiota regulates host metabolism, immunity, and nervous system. Additionally, gut microbiota is associated with cancer development [8–11]. Recent studies have demonstrated that *Helicobacter pylori* is a risk factor for gastric cancer [12]. The abundance of *Faecalibacterium prausnitzii* and *Blautia* sp. is correlated with the malignancy of breast cancer [13]. Studies on the tumor-related pathway have revealed that gut microbiota can activate the calcineurin-NFAT pathway, promoting intestinal tumor development and supporting cancer stem cell survival in the mouse model [14]. Butyrate, which is produced by butyrate-producing intestinal bacteria, activates Gpr109a and suppresses colonic inflammation and carcinogenesis [15]. However, the correlation of gut microbiota and gut microbiota-related metabolites with OS has not been previously reported.

The gut microbiota composition is dynamic and is mainly influenced by various factors, including age, eating habits, and health and disease status, which modulate the diversity and metabolites of microorganisms. Additionally, variations in the gut microbiota metabolic spectrum promote physiological changes in both host and pathogenic microorganisms and consequently modulate disease progression [16]. Some metabolism and metabolic profiling studies have reported that metabolites are potential diagnostic markers for diseases [17]. Previous studies using the 16S rRNA gene sequencing approach have revealed that microbiome diversity and metabolites are correlated with disease pathogenesis. For example, enterogenous *Candida albicans* damages the intestinal mucosal barrier by modulating the gut microbiome [18]. Meanwhile, 3-carboxy-4-methyl-5-propyl-2-furanpropionic acid was identified in the plasma of patients with gestational diabetes, impaired glucose tolerance, and type 2 diabetes [19]. However, the OS-related microbiome composition and metabolites have not been previously reported.

This study aimed to evaluate the effects of OS on intestinal microbes and their metabolites. The fecal samples of the OS mouse model were examined to evaluate the changes in gut microbiota and gut microbiota-derived metabolites using 16S rRNA high-throughput sequencing and liquid chromatogram-mass spectrometry (LC-MS)-based non-targeted metabolomics, respectively. Additionally, the distribution of intestinal microbes and their metabolites, as well as the correlation between OS and intestinal microbes, were examined. The findings of this study can enable the development of probiotics and the identification of beneficial metabolites for OS.

## MATERIALS AND METHODS

### Cell culture

The human OS cell line (Saos-2) was obtained from the American Type Culture Collection (Manassas, VA, USA) and cultured in Dulbecco’s modified Eagle’s medium-F12 with low glucose (Gibco, USA) supplemented with 10% fetal bovine serum, 100 U/mL penicillin and 100 lg/mL streptomycin in a humidified atmosphere at 5% CO_2_ and 37°C.

### Animal experiments

**Figure 1:**
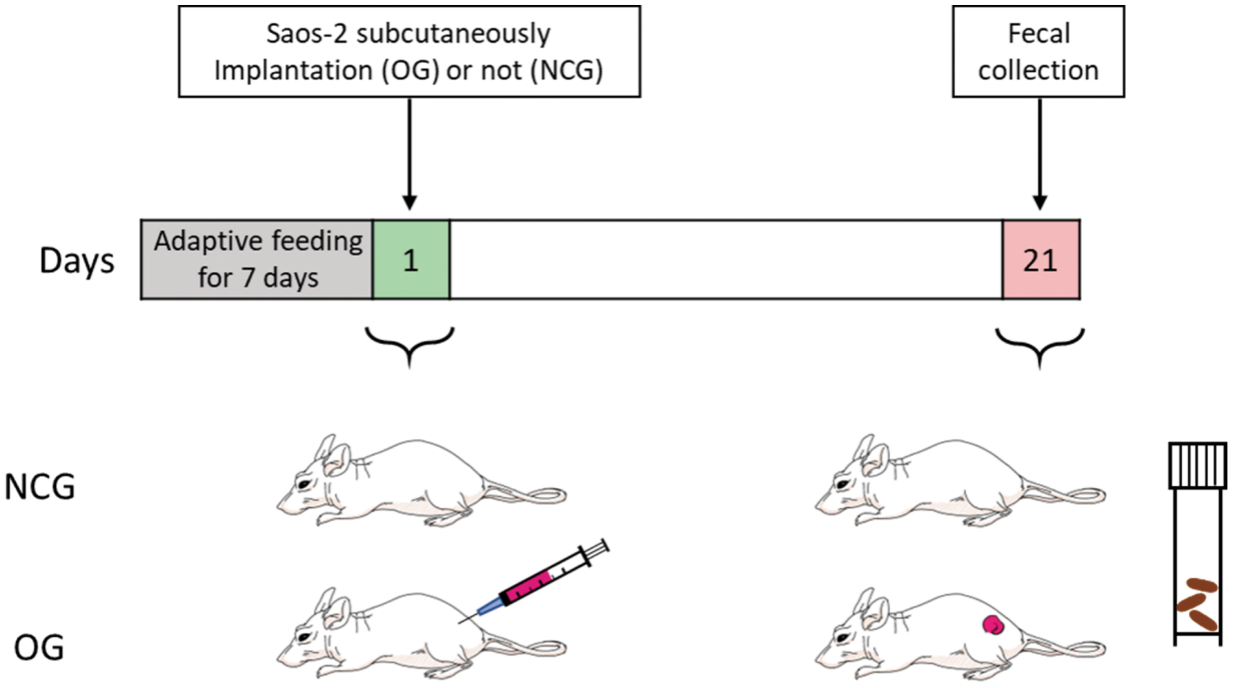
**Construction of the osteosarcoma mouse model with Saos-2 OS cells.** NCG represents the control group, OG represents the osteosarcoma group. n = 7.

Female BALB/c nude mice aged 4 weeks were purchased from a specific pathogen-free (SPF) animal center (Charles River Laboratory, Beijing, China). Mice were maintained at the SPF animals center under the following standard laboratory conditions: temperature, 25°C ± 3°C; humidity, 53% ± 3%, circadian cycle, 12-h light/dark cycle; access to food and water, ad libitum. After adaptive feeding for 1 week, 14 mice were randomly divided into the following two groups (7 mice/group): NCG and OG. Nude mice aged 5 weeks in the OG were subcutaneously implanted with 1 × 10^6^ Saos-2 cells mixed with 200 μL Matrigel (Corning, NY, USA) into the back flank of each mouse. Meanwhile, mice in the NCG were administered with 200 μL Matrigel (Corning, NY, USA) at the same site. The tumor exhibited good growth in the OG at week 2 post-administration. The OS animal model was successfully established at week 3 post-administration (Figure 1).

### Fecal sample collection

The fresh fecal samples of the NCG and OG were collected and rapidly stored at −80°C in sterile freezing tubes. Next, the fecal samples were subjected to 16S rRNA gene sequencing and untargeted metabolomics analysis. All mice were anesthetized via CO_2_ inhalation and humanely euthanized.

### DNA extraction and 16S rRNA gene sequencing

The fecal samples from the NCG and OG were subjected to 16S rRNA sequencing and metabolomics analyses. The genomic DNA was extracted from the fecal samples using the MagPure soil DNA LQ kit (Magen, Guangdong, China), following the manufacturer’s instructions. DNA concentrations and integrities were determined using a NanoDrop 2000C spectrophotometer (Thermo Fisher Scientific, Waltham, MA, USA) and gel electrophoresis. To analyze fecal bacterial diversity, V3–V4 hypervariable regions of the bacterial 16S rRNA genes were amplified with the universal primers 343 F (5′-TACGGRAGGCAGCAG-3′) and 798 R (5′-AGGGTATCTAATCCT-3′). The reverse primer comprised a sample barcode, while both forward and reverse primers were ligated with an Illumina sequencing adapter. Sequencing was performed using an Illumina NovaSeq6000 platform with two paired-end read cycles of 250 bases each (Illumina Inc., San Diego, CA, USA; OE BioTech Company; Shanghai, China). The quality of amplicons was tested, and the raw data were obtained in the FASTQ format. The assembly parameters were as follows: minimum overlap, 10 bp; maximum overlap, 200 bp; maximum error ratio, 20%. Homologous sequences with a size of < 200 bp were removed, whereas sequences with 75% of the base readings above Q20 were retained. Clean tags were removed using UCHIME [20] to obtain valid tags for preparing operational taxonomic units (OTUs). After removing the primer sequences and clustering with a cutoff value of 97% similarity, the OTUs were classified using Vsearch software (version 2.4.2) [21]. The QIIME package was used to select the representative reading of each OTU. The species of all representative reads above the confidence threshold of 70% were annotated using the RDP classifier with the Silva database (version 123) [22].

### Metabolomic data processing and analysis

The fecal samples from the NCG and OG were subjected to metabolomics analysis, which was performed by OE BioTech (Shanghai China). Each fecal sample (50 mg) was mixed with 500 μL of extraction solvent (methanol/water 4:1 ratio, v/v) and 40 μL of internal standard solution (2-chloro-L-phenylalanine in methanol, 3 g/L) in a 2-mL microcentrifuge tube, and the mixture was sonicated at 60 Hz for 3 min. Next, the samples were incubated with 120 μL of chloroform, vigorously vortexed, and subjected to ultrasonic extraction at 25°C for 20 min. The samples were then centrifuged at 13,680 *g* and 4°C for 20 min. The supernatant was dried under vacuum for 30 min at 25°C and dissolved in 80 μL methoxyamine hydrochloride in pyridine (15 mg/μL). After vigorously vortexing for 10 min, the products were incubated at room temperature for 80 min, followed by incubation with 20 μL of n-hexane and 60 μL of N, O-bis(trimethylsilyl)trifluoroacetamide (containing 2% trimethylchlorosilane), vigorous vortexing for 3 min, and derivatization at 65°C for 70 min. The results were analyzed using a gas chromatography system (Agilent 7890 B System) coupled to the Agilent 5977 A MSD System (Agilent Technology, Santa Clara, CA, USA). Based on the standard protocol, the derivatives were separated using a DB-5MS fused silica capillary column (30 mm × 0.25 mm × 0.25 μm) (Agilent Technology, USA). Mass spectrometry data (m/z 50–500) were obtained under the full-scan mode. To check the data reproducibility, quality control samples were injected regularly throughout the analysis process [23, 24].

### Untargeted metabolomics data analysis

The metabolome raw data were collected using Unifi 1.8.1 and processed using the Progenesis Qi V2.3 software (Nonlinear Dynamics, Newcastle, UK). The compounds were identified using The Human Metabolome Database, Lipidmaps (V2.3), METLIN databases, and self-built databases based on the accurate mass number, secondary fragments, and isotope distribution. The compounds were qualitatively screened according to the screening standard of 36 points. Compounds with < 36 points were considered to be inaccurate and deleted (full score = 60 points). Principal component analysis (PCA) and orthogonal partial least squares discriminant analysis (OPLS-DA) were performed to examine the differential metabolic profiles between the two groups. The Hotelling’s T2 region demonstrated an ellipse shape in the model score, which was defined at a 95% confidence interval for model variation. In OPLS-DA, variable importance in projection (VIP) was employed to measure the influence and explanatory ability of the samples in each group. A VIP score of > 1 was considered the cutoff value. Differential metabolites were selected according to the threshold of significant variables obtained from the OPLS-DA model based on VIP values and p-values obtained from two-tailed Student’s t-test of normalized peak areas. Metabolites with VIP values > 1.0 and p < 0.05 were considered to be differential metabolites.

### Statistical analysis

Means were compared using Student’s t-test with SPSS 22.0 software. The levels of the gut microbiota and metabolites were analyzed using the Wilcoxon test, Bray-Curtis distance, Euclidean distance, and Unifrac and presented as mean ± standard error of mean. Differences were considered significant at P < 0.05. The correlation between the gut microbiome and metabolites was analyzed based on Pearson’s correlation coefficients.

## RESULTS

### Alterations of the diversity and abundance of gut microbiota species in the OS mouse model

**Figure 2:**
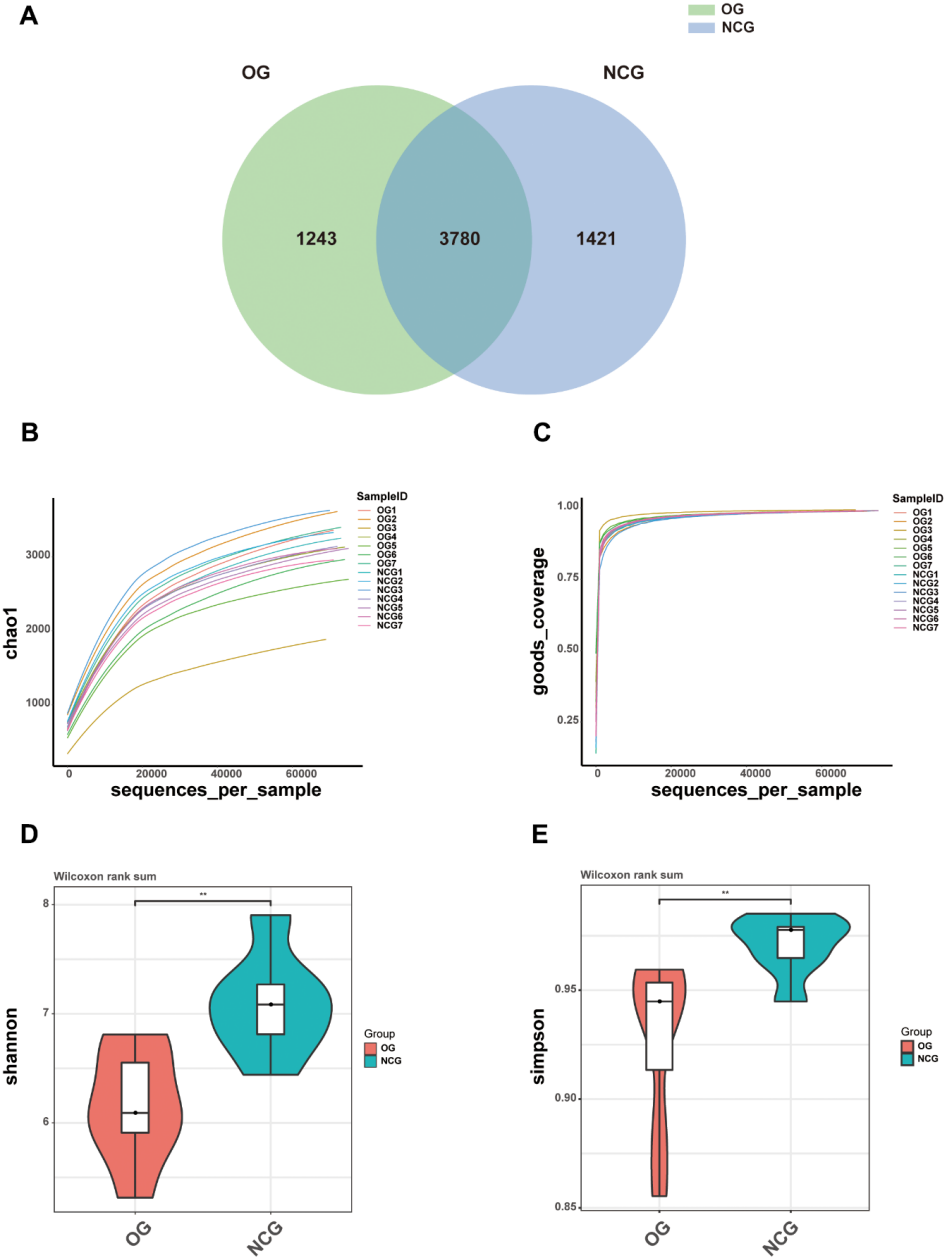
(**A**) Venn diagram showing the numbers of operational taxonomic units (OTUs) between NCG and OG. (**B**–**E**) Alpha diversity of samples from the NCG and OG groups. (**B**) Rarefaction curves with Chao1 index. (**C**) Rarefaction curves with Good’s coverage index. (**D**) Violin plot of Shannon index. (**E**) Violin plot of Simpson index. Data are represented as mean ± standard error of mean. n = 7; ^*^P < 0.05, ^**^P < 0.01, ^***^P < 0.001, and ^****^P < 0.0001; ns, non-significant.

**Figure 3:**
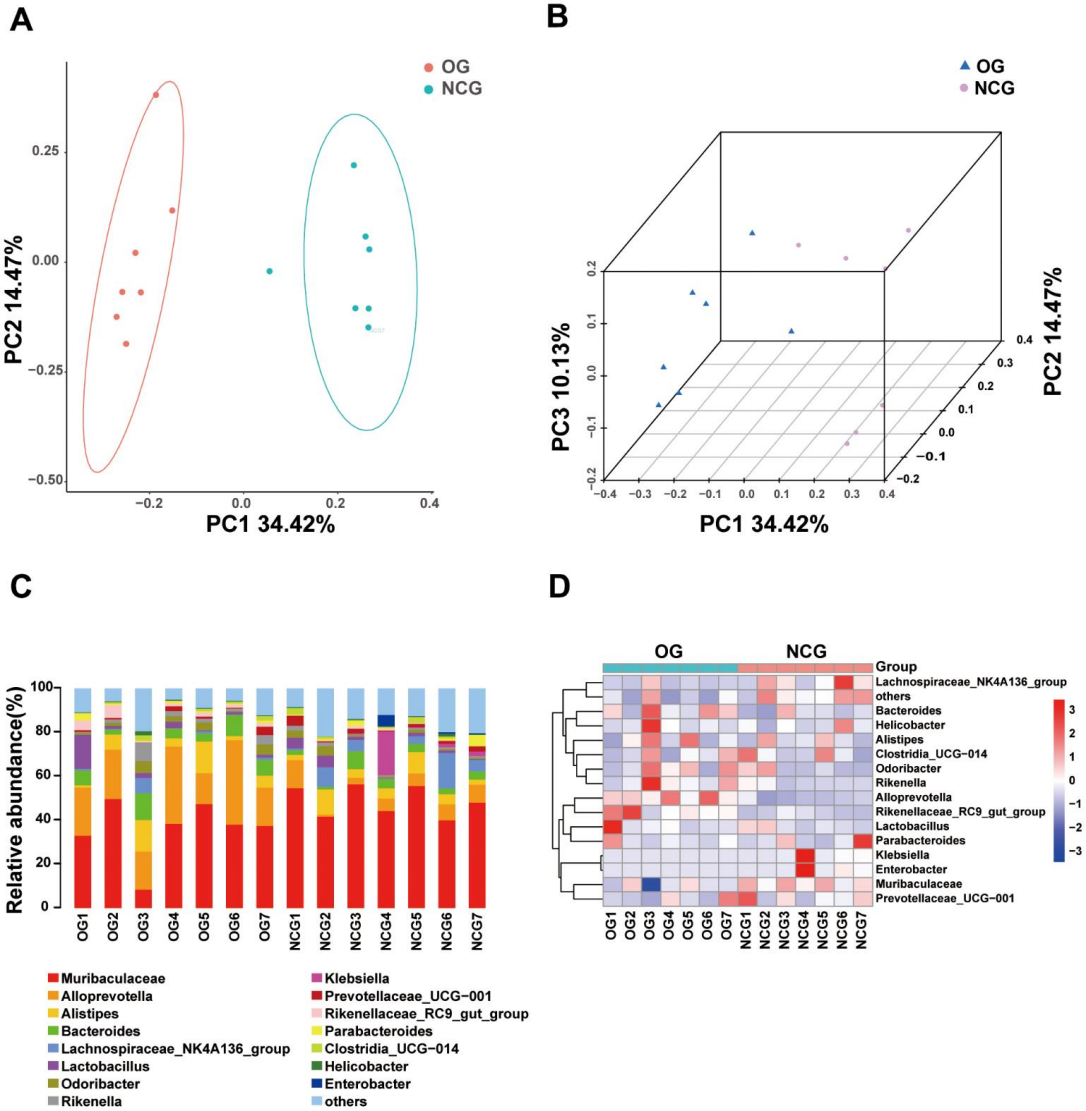
(**A**) Two-dimensional principal coordinate analysis (PCA) model of gut microbiota. NCG: green, OG: orange. (**B**) Two-dimensional PCA of gut microbiota. NCG: purple, OS: blue. (**C**) Stacked bar chart of the abundance of microbes at the *genus* level. (**D**) Heatmap of the abundance of microbes at the *genus* level. n = 7.

This study performed 16S rRNA high-throughput gene sequencing to examine the effect of OS on the gut microbiota. Venn diagram of the OTU distribution revealed changes in the microbiota composition in the OG. In total, 6,444 OTUs were identified in the NCG and OG. Of these, the NCG and OG had 1,421 and 1,243 unique OTUs, respectively. Additionally, 3,780 and 1291 were shared and differential OTUs, respectively, between the groups (Figure 2A and Supplementary Table 1). Alpha diversity analysis was performed to evaluate community diversity and abundance in gut microbiota. Rarefaction curve, Chao1 index, and Good’s coverage index revealed that the sequencing depth was sufficient and could provide coverage of the majority of microbiota diversity in each sample (Figure 2B, 2C). Compared with those in the NCG, the Shannon and Simpson diversity indices were lower in the OS. This indicated that OS decreased the bacterial community diversity and richness (P < 0.05) (Figure 2D, 2E). Next, beta diversity, which reflects differential species abundance between the two groups, was examined. Two-dimensional (2D) and three-dimensional (3D) PCA revealed that the gut microbiota of the NCG and OG separated into two distinct clusters, indicating the differential gut microbiota composition between the NCG and OG (P < 0.05) (Figure 3A, 3B).

**Figure 4:**
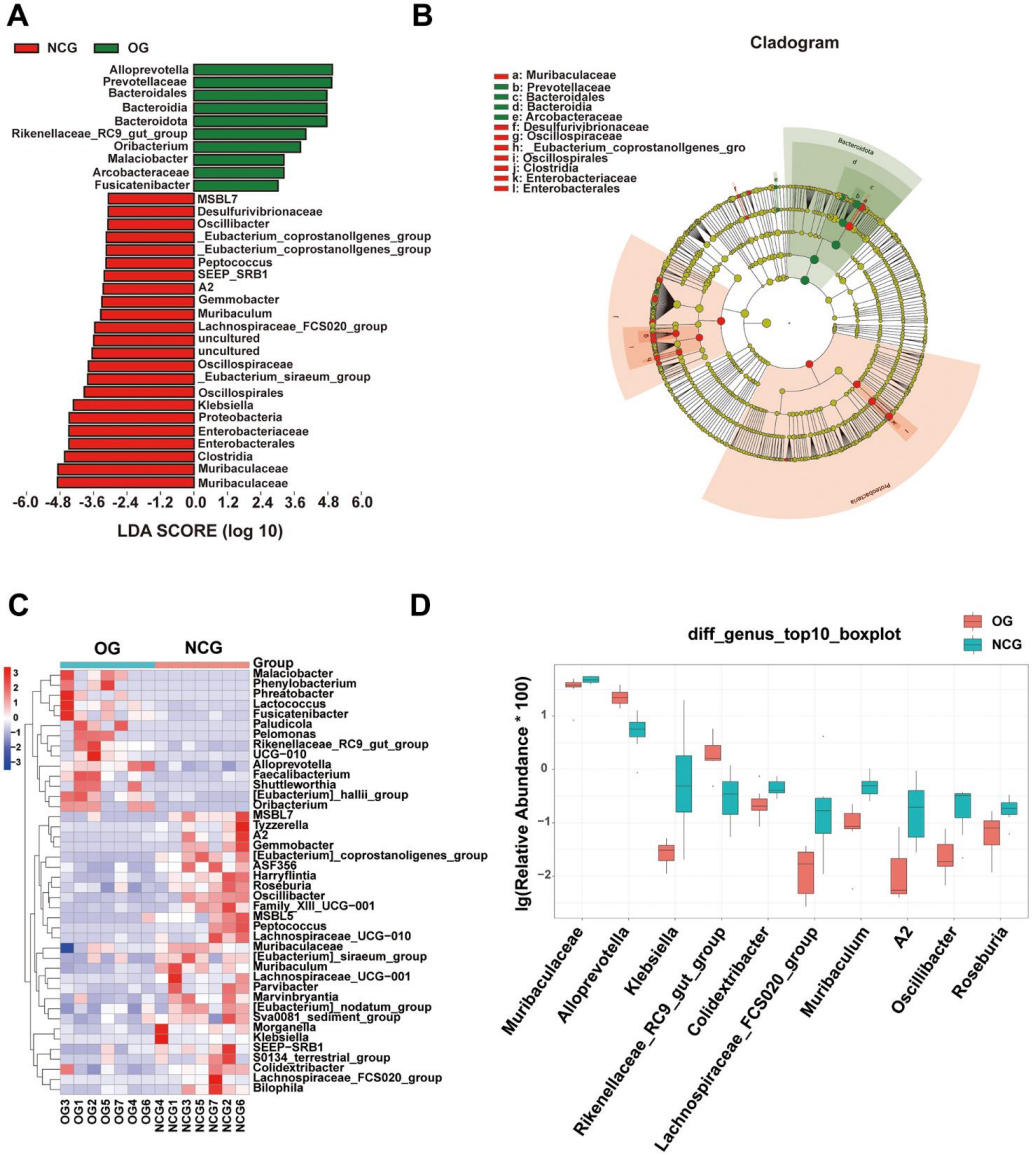
(**A**) Linear discriminant analysis effect size of gut microbiota in the NCG and OG. Red represents increased microbial abundance in the NCG; green represents increased microbial abundance in the OG. (**B**) The correlations among different taxa from the *phylum* to *species* levels are shown in the cladogram. (**C**) Heatmap showing differential bacterial genera. (**D**) Histogram of the top 10 differential bacteria at the *genus* level. Wilcoxon test (P < 0.05; n = 7).

Stacked bar charts and heatmaps of the top 15 *phylum*, *classes*, *orders*, *families*, *genus*, and *species* were generated to perform a species analysis. The *genus*-level analysis result is shown in Figure 3C, 3D, while the results of analysis at other levels are shown in Supplementary Figure 1. Further, a linear discriminant analysis effect size analysis was performed to assess the significant differences in the abundance of the species between the two groups. The correlation between different taxa from the *phylum* to the *species* levels is shown in the cladogram in Figure 4A, 4B. The histograms and heatmaps of abundances at the *genus* level are shown in Figure 4C, 4D. Among the top 10 differential genera between the NCG and OG, *Alloprevotella* and *Rikenellaceae_RC9_gut_group* were upregulated, whereas *Muribaculaceae*, *Klebsiella*, *Colidextribacter*, *Lachnospiraceae_FCS020_group*, *Muribaculum*, *A2*, *Oscillibacter*, and *Roseburia* were downregulated.

### OS altered the composition of gut microbiota-derived metabolites

This study aimed to examine the effects of OS on intestinal microbes and their metabolites. Changes in the metabolic spectrum may indicate changes in the dynamics of the intestinal microbiome. Metabolic changes are correlated with the host’s health status and are considered a crucial hallmark of disease [25]. To evaluate the effect of OS on the metabolic profiles of gut microbiota in the mouse model, untargeted metabolomics analysis (LC-MS and GC-MS) was performed by OE BioTech (Shanghai, China) to detect differentially expressed metabolites and potential key metabolic pathways in the NCG and OG.

**Figure 5:**
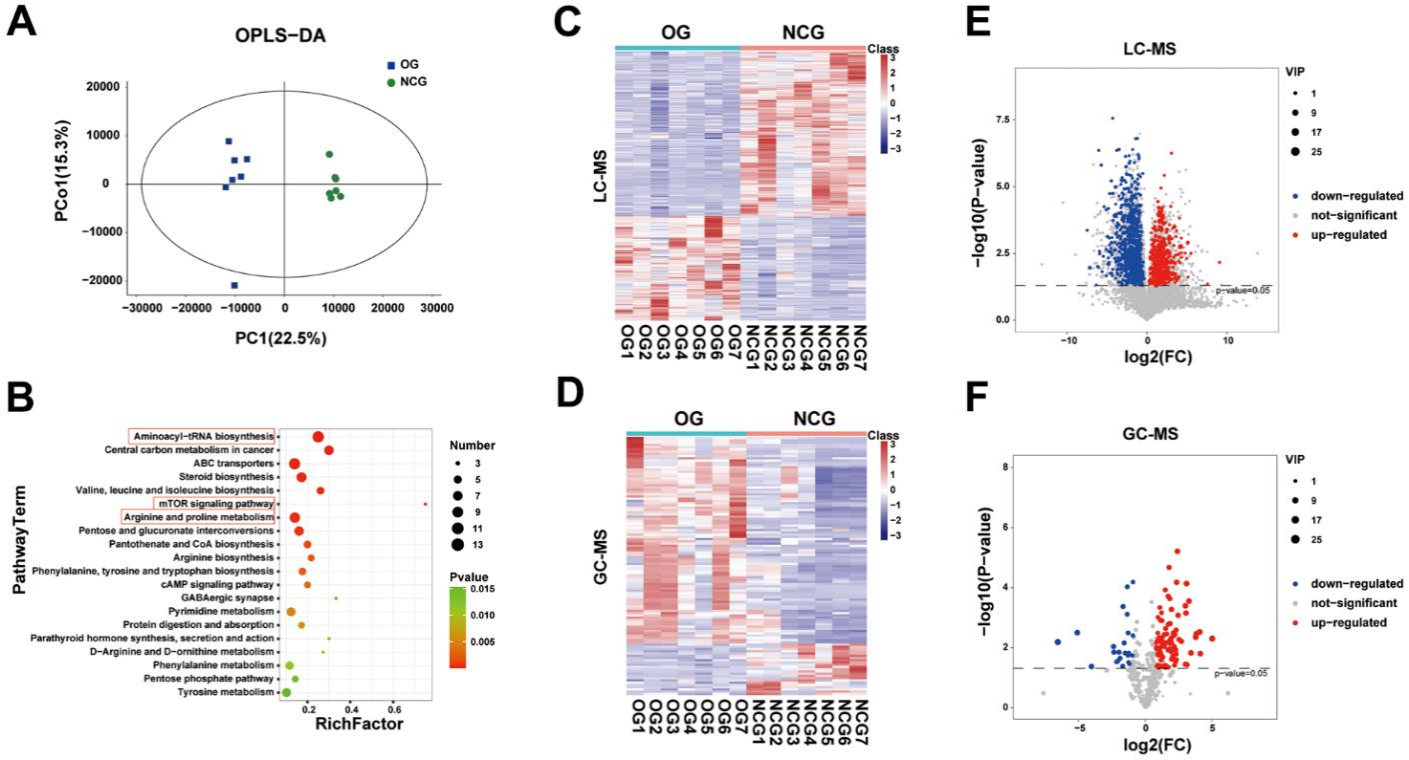
(**A**) Orthogonal partial least squares discriminant analysis (OPLS-DA) of fecal metabolites between the NCG and OG. (**B**) Bubble diagram of the top 20 enriched Kyoto Encyclopedia of Genes and Genomes (KEGG) pathways. (**C**) Heatmap of fecal metabolites identified using gas chromatography-mass spectrometry (GC-MS). (**D**) Heatmap of fecal metabolites identified using liquid chromatography-mass spectrometry (LC-MS). (**E**) Volcano map displaying differential fecal metabolites identified using GC-MS. (**F**) Volcano map displaying differential fecal metabolites identified using LC-MS. n = 7.

Multivariate analysis utilizes unsupervised PCA to examine the overall distribution between samples and the stability of the whole analysis process. Next, supervised PLS-DA and OPLS-DA were performed to distinguish the overall differences in metabolic profiles among the groups and identify the differential metabolites between the groups. Two-dimensional and three-dimensional PCA, PLS-DA, and OPLS-DA were performed to identify the differential metabolic profiles between the two groups (Figure 5A). Univariate analysis focuses on the description of univariate and statistical inference, which are used to select differential metabolites, and reflects the basic information contained in a large number of sample data in the simplest summary form. Additionally, the centralized or discrete trend in the sample data is described. The univariate statistical inference provides the overall information of the sample data, mainly including interval estimation and statistical hypothesis testing. Volcano maps and heatmaps were used to visualize the differential metabolites (Figure 5C–5F). The volcano diagram revealed different levels of metabolites in the two groups. LC-MS revealed 1094 differential metabolites (424 upregulated and 670 downregulated metabolites) between the two groups, while GC-MS revealed 115 differential metabolites (92 upregulated and 23 downregulated metabolites). The differential fecal metabolites between the two groups were subjected to Kyoto Encyclopedia of Genes and Genomes (KEGG) enrichment analysis. The top 20 differential metabolites are listed in Figure 5B. This study focused on the following three metabolic pathways associated with bone metabolism: aminoacyl-tRNA biosynthesis; mTOR signaling pathway; arginine and proline metabolism. The metabolites of these three pathways are shown in Supplementary Table 2.

### Correlation between differential gut microbial genera and fecal metabolome

**Figure 6:**
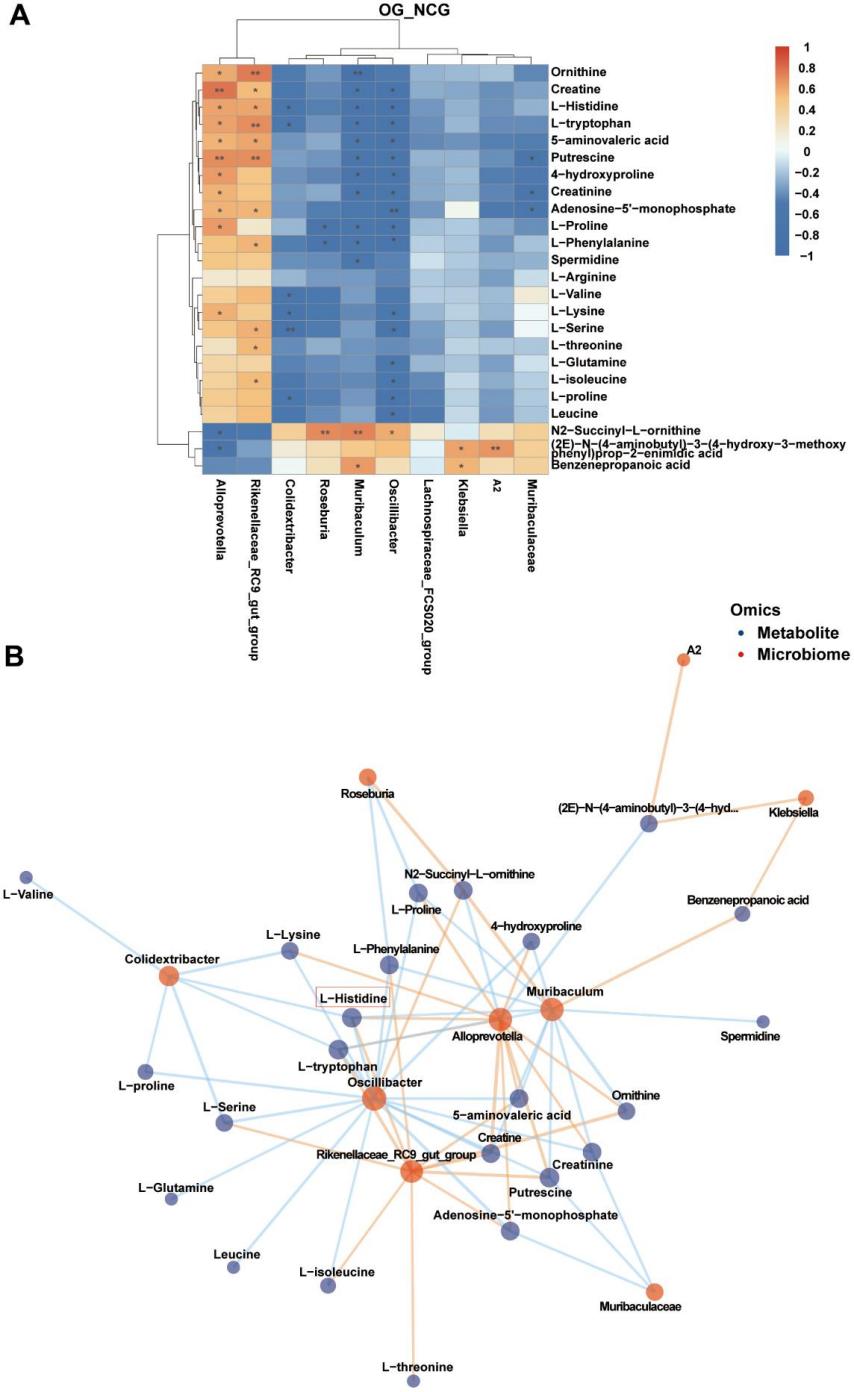
(**A**) Correlation heatmap between the top 10 differential bacterial genera and fecal metabolites associated with three metabolic pathways related to bone metabolism in the top 10 Kyoto Encyclopedia of Genes and Genomes (KEGG) pathways. |r| > 0.5, P < 0.05. (**B**) Correlation network map of the top 10 differential bacterial genera and fecal metabolites associated with three metabolic pathways related to bone metabolism in the top 10 KEGG pathways. |r| > 0.5, P < 0.05. n = 7.

The correlation heatmap and network of fecal differential metabolites in the three bone metabolism-related metabolic pathways and the top 10 differential genera are shown in Figure 6A, 6B (|r| > 0.5, P < 0.05). The analysis revealed multiple correlations between these fecal differential metabolites and the top 10 differential bacterial genera. For example, *Alloprevotella* was positively related with creatine (r = 0.778, P = 0.001), putrescine (r = 0.691, P = 0.006), L-proline (r = 0.649, P = 0.012), 4-hydroxyproline (r = 0.642, P = 0.013), L-tryptophan (r = 0.624, P = 0.017), L-histidine (r = 0.613, P = 0.019), ornithine (r = 0.599, P = 0.024), L-lysine (r = 0.576, P = 0.031), creatinine (r = 0.565, P = 0.035), adenosine-5′-monophosphate (r = 0.561, P = 0.037), and 5-aminovaleric acid (r = 0.561, P = 0.037). *Rikenellaceae_RC9_gut_group* was positively correlated with ornithine (r = 0.759, P = 0.002), putrescine (r = 0.698, P = 0.006), L-tryptophan (r = 0.690, P = 0.006), 5-aminovaleric acid (r = 0.608, P = 0.021), L-histidine (r = 0.605, P = 0.022), L-serine (r = 0.576, P = 0.032), adenosine-5’-monophosphate (r = 0.560, P = 0.037), L-phenylalanine (r = 0.552, P = 0.041), L-threonine (r = 0.549, P = 0.041), L-isoleucine (r = 0.537, P = 0.048), and creatine (r = 0.533, P = 0.049). *Muribaculum* was negatively correlated with ornithine (r = −0.723, P = 0.003), 4-hydroxyproline (r = −0.621, P = 0.018), L-phenylalanine (r = −0.594, P = 0.025), putrescine (r = −0.593, P = 0.025), creatine (r = −0.592, P = 0.026), L-tryptophan (r = −0.581, P = 0.029), creatinine (r = −0.579, P = 0.030), L-histidine (r = −0.551, P = 0.041), L-proline (r = −0.545, P = 0.044), spermidine (r = −0.539, P = 0.047), and 5-aminovaleric acid (r = −0.535, P = 0.048). These results suggest that the occurrence and development of OS are closely related to amino acid metabolism.

## DISCUSSION

The number of genes encoded by the gut microbiota, which comprises the intestinal commensal, symbiotic, and pathogenic microorganisms, is more than 3 million, which is approximately 150 times higher than that encoded by the host genome. The gut microbiota regulates various functions of the host and consequently influences host health, phenotype, and diseases [26, 27]. Recently, examining the role of GM in various diseases has become a research hotspot. Dysbiosis is associated with several complex human intestinal and extra-intestinal diseases, obesity, diabetes, non-alcoholic liver disease, cardiovascular disease, malnutrition, gastrointestinal diseases, depression, Parkinson’s disease, and cancers [25, 28, 29]. Gut microbiota is involved in the development and progression of cancers [30]. Extra-intestinal tumor-associated microbiota has been identified in multiple human cancers, including breast, lung, and pancreatic cancers, melanoma tumors, and soft tissue sarcoma [31, 32]. Alice Tzeng et al. characterized the microbiome of human breast tissue. The breast tumor tissue exhibited distinct microbiome profiles, including increased proportions of *Pseudomonas*, *Proteus*, *Porphyromonas*, and *Azomonas* and decreased proportions of *Propionibacterium* and *Staphylococcus* at the *genus* level [33]. Erick Riquelme et al. examined the gut microbiota of short-term and long-term survivors among patients with pancreatic cancer who underwent tumor resection. The alpha diversity was upregulated in the long-term survivors. Additionally, an intra-tumoral microbiome signature that can predict long-term survival was identified. Deborah Nejman et al. revealed that the abundance of *Proteobacteria phylum* was upregulated in the lung tumors of smokers. Additionally, the authors reported that different tumor types exhibit distinct microbial composition [34]. Recently, Lauren M Perry et al. used metagenomic classification to investigate the gut microbiome in patients with soft tissue sarcoma [35]. However, the gut microbiota and gut microbiota-derived metabolites have not been elucidated for OS, which is the most common bone sarcoma.

Animal models, which cannot be replaced by *in vitro* models, are an important component of translational research [36]. Athymic nude mice, which are widely used as models in cancer research, bear spontaneous FOXN1 deletion and have dysfunctional or absent thymus, resulting in an impaired immune system with downregulation of T cells [37]. In this model, natural immunosuppression enables the development of the target tumor after the inoculation of cancer cells. Additionally, the tumor can be easily observed in athymic nude mice after subcutaneous injection due to the natural lack of hair [37]. In addition to analyzing tumor behavior, athymic mice have been used to examine the gut microbiome and its relation to cancer [32]. Therefore, this study established an athymic nude mouse model of OS to examine the abundance of gut microbiota and gut microbiota-derived metabolites and develop novel therapeutic strategies for OS.

Microbial diversity was evaluated using 16S rDNA sequencing, which enables high-throughput sequencing of all bacteria in a particular environmental sample to explore the correlation between the microbe and the host. Traditional microbial research relies on laboratory culture. The recent development of 16S amplicon sequencing and other high-throughput sequencing methods has filled the gap in microbial research, especially for microorganisms that cannot be cultured in traditional laboratories, and expanded the utilization space of microbial resources. These sequencing methods are effective for studying microbial interaction. Alpha and beta diversity analyses are the two main components of 16S rDNA sequencing. Alpha diversity indicates the diversity within a particular environment or ecosystem and is primarily used to reflect species richness and evenness, as well as sequencing depth [38]. Beta diversity indicates the similarity or dissimilarity among different environmental communities [39]. In this study, 16S rDNA sequencing analysis revealed that the beta diversity of gut microbiota varied between the NCG and OG. Additionally, alpha diversity analysis revealed that the richness and diversity of gut microbiota were downregulated in the OG. The relative abundance of the genera *Alloprevotella* and *Rikenellaceae_RC9_gut_group* was upregulated, whereas that of *Muribaculum* was downregulated in the OG. The abundance of *Alloprevotella* is reported to be upregulated in multiple cancers. Wu et al. reported that *Alloprevotella* was associated with an increased risk of cardia cancer [40]. Wei et al. investigated the correlation between bacterial profiles and the symptoms of pancreatic adenocarcinoma (PDCA). The abundance of *Alloprevotella* was upregulated in patients with PDCA exhibiting bloating [41]. Wang et al. developed an ulcerative colitis (UC) carcinogenesis mouse model. The abundance of *Alloprevotella* was upregulated in the intestinal mucosa of the UC mouse model [42]. Moreover, the relative abundance of the genera *Alloprevotella* was upregulated in various cancers, including breast, thyroid, colorectal, and oral cancers [43–46]. *Rikenellaceae_RC9_gut_group* is also reported to be associated with the pathogenesis of some cancers. The relative abundance of *Rikenellaceae* was significantly upregulated in patients with prostate cancer belonging to the high-risk group [47]. Yang et al. reported that the abundance of *Rikenellaceae_RC9_gut_group* was significantly upregulated in proximal gastric cancer tissues and positively correlated with cancer-promoting metabolites [48]. Previous studies have reported that *Rikenellaceae_RC9_gut_group* is associated with inflammation [49]. Cancer development and therapy response are regulated by inflammation, which may facilitate tumor progression and treatment resistance [50]. *Muribaculum*, which is associated with improved response to immunotherapy, is a cancer-related bacterium [51]. Zhao et al. used a combination of probiotics and PD-1 inhibitors to treat melanoma. The authors reported that the beneficial bacteria *Akkermansia*, *Prevotellaceae_NK3B31_group*, and *Muribaculum* were enriched in the combination treatment group [52]. However, in this study, the relative abundance of *Muribaculum* in the OG was significantly lower than that in the NCG. These findings suggest that the increased abundance of *Alloprevotella* and *Rikenellaceae_RC9_gut_group* and the decreased abundance of *Muribaculum* are associated with OS pathogenesis.

Dysregulated metabolism is a hallmark of cancer [53]. To elucidate the regulatory effects of gut microbiota on OS pathogenesis, the metabolome was examined to determine the pathogenic mechanism of OS. Metabolomics is an emerging omics discipline that can identify and quantify most small molecules in living organisms [54]. As metabolites exhibit a wide range of functions in the cells and organisms and reflect the overall effect of the genome, proteome, and external stimuli, the metabolome can indicate the phenotype [55]. Metabolomics is now widely used in many fields, such as environmental science and food safety [56]. Based on research applications, metabolomics can be divided into non-targeted metabolomics and targeted metabolomics. Several analytical techniques are now used in non-targeted metabolomics, including nuclear magnetic resonance spectroscopy, GC-MS, and LC-MS [57–59]. In this study, GC-MS and LC-MS (dual mode) were used to comprehensively screen the differential metabolites. Fecal metabolomics revealed the differential metabolites between the NCG and OG. Next, KEGG enrichment analysis of the differential fecal metabolites between the two groups was performed. The metabolites of three bone metabolism-related metabolic pathways were annotated. Amino acid metabolism had an important role in OS pathogenesis, which is consistent with the findings of a previous study [60].

In addition to their role in protein synthesis, amino acids play an important role in energy production, nucleotide synthesis, and the maintenance of redox homeostasis [61, 62]. Early studies on cancer metabolism focused on glucose metabolism. Recent studies have demonstrated the importance of amino acids in cancer progression [63]. Amino acid-derived metabolites support cancer growth and metastasis. The catabolism of amino acids results in the production of metabolic intermediates that regulate tumor cell growth and survival [64]. Additionally, amino acids modulate reactive oxygen species homeostasis and are involved in epigenetic regulation through methylation and acetylation, which can enhance tumor aggressiveness [64]. Amino acids are reported to be involved in cancer metabolism. However, limited studies have examined the metabolomic changes in OS. Recently, the elucidation of the role of amino acids in primary bone sarcomas has become the focus of research. Phosphoglycerate dehydrogenase inhibition can lead to the upregulation of SLC7A5 (LAT1) and SLC3A2 (CD98) transporters that drive the transport of leucine into the lysosome, resulting in mTORC1 activation [65]. Previous studies have demonstrated that mTORC1 regulates OS cell proliferation partly by modulating serine/glycine metabolism [66]. In this study, the mTOR signaling pathway and amino acid metabolism were dysregulated in the OG. Therefore, these results suggest that amino acid metabolism may play a vital role in the carcinogenesis of OS. To further examine the crosstalk between gut microbiota and metabolites, correlation analysis was performed. For example, L-histidine was associated with *Alloprevotella*, *Rikenellaceae_RC9_gut_group*, and *Muribaculum* to varying degrees. A previous study reported that the serum contents of metabolites, especially histidine, markedly varied between the OS and healthy control groups [67]. Previously, we reported that histidine metabolism was upregulated in OS 3D cells and 3D cell-printed tissues [60]. Hence, histidine metabolism is a key pathway involved in OS progression.

This study has some limitations. The sample size in this study is relatively small. Studies must be performed with a large sample size to identify an ideal biomarker for OS. Additionally, this study examined the correlation between the gut microbiota of the OG and metabolites but did not establish a causal relationship.

## CONCLUSIONS

This study demonstrated that gut microbiota and gut microbiota-related metabolites are correlated with OS. Thus, gut microbiota plays an important role in the pathogenesis of OS. These findings provided novel insights into the pathogenesis of OS and can enable the development of novel therapeutic strategies for OS.

## Supplementary Material

Supplementary Figure 1

Supplementary Table 1

Supplementary Table 2
