# Supplementary material for: Characterization of the gut microbiota and fecal metabolome in the osteosarcoma mouse model
Source: Aging (Albany NY). 2024 Jul 3;16(13):10841–59. doi: 10.18632/aging.205951 (PMC11272122; doi:10.18632/aging.205951)
Supplement: Supplementary Figure 1 [file aging-16-205951-s001.pdf]

SUPPLEMENTARY FIGURE

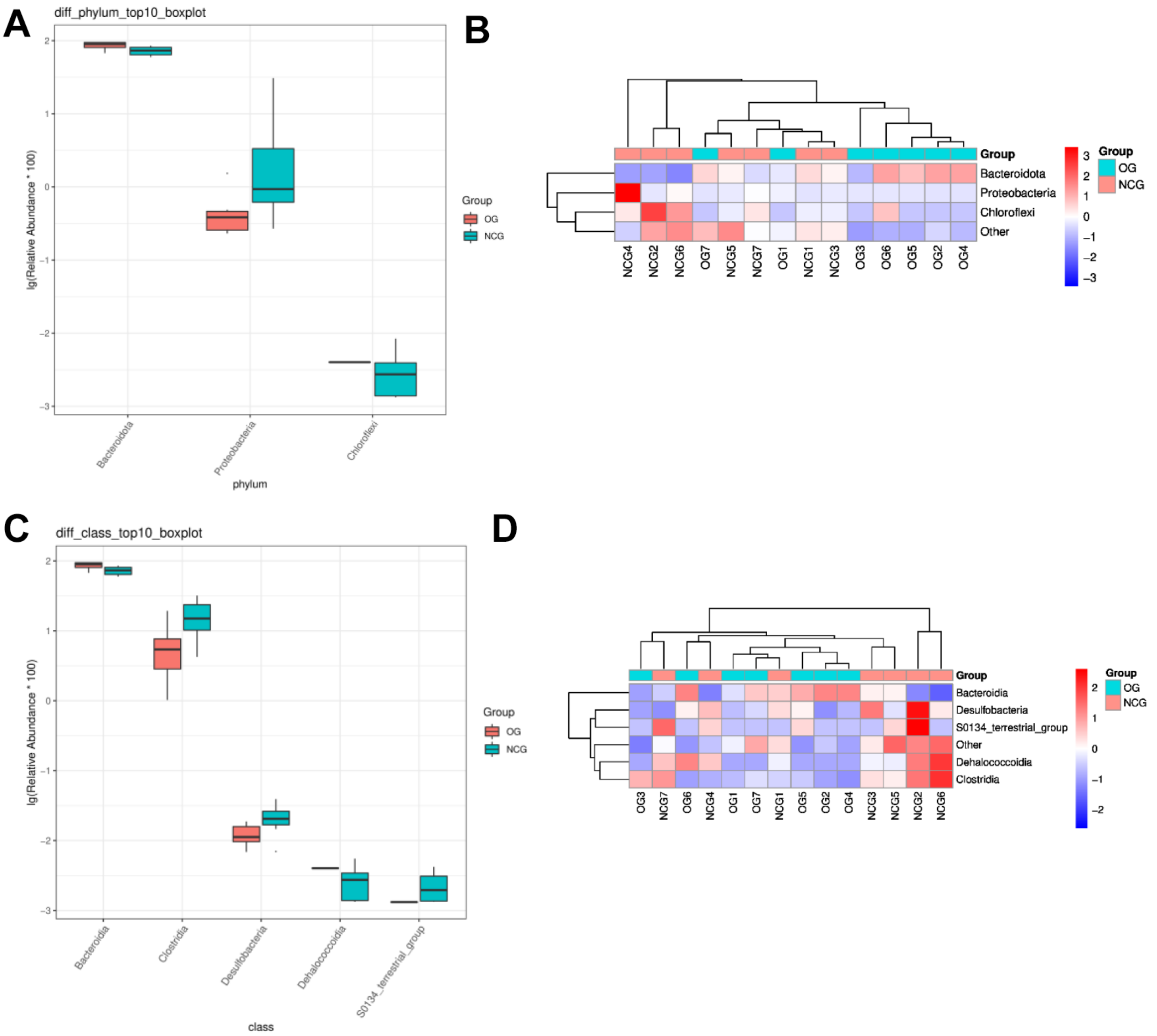

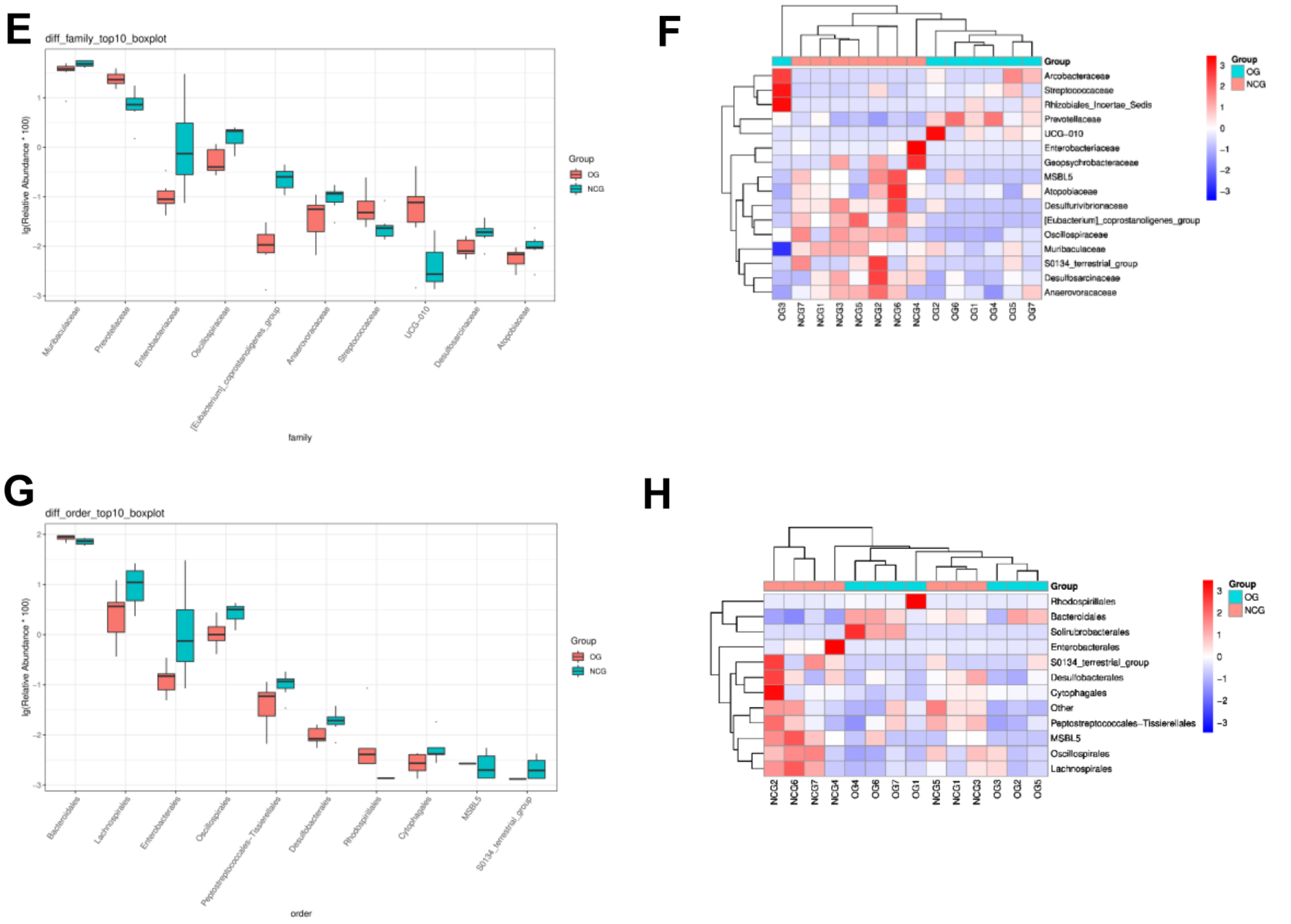

Supplementary Figure 1. The stacked bar charts and heat maps of other levels between the two groups. (**A**, **B**) *phylum*-level. (**C**, **D**) *class*-level. (**E**, **F**) *family*-level. (**G**, **H**) *order*-level. n = 7.
