# Supplementary material for: Characterization of the gut microbiota and fecal metabolome in the osteosarcoma mouse model
Source: Aging (Albany NY). 2024 Jul 3;16(13):10841–59. doi: 10.18632/aging.205951 (PMC11272122; doi:10.18632/aging.205951)
Supplement: Supplementary Table 1 [file aging-16-205951-s002.docx]

**Supplementary Table 1. Differential OTUs calculated by Wilcoxon.**

| OUT_ID | Test | p-value | FDR_P | Bonferroni_P | OG_mean | NCG_mean |
| --- | --- | --- | --- | --- | --- | --- |
| 1 | 0 | 0.000999 | 0.050695 | 1 | 512 | 10.42857 |
| 9 | 0 | 0.000999 | 0.050695 | 1 | 1735.286 | 65.71429 |
|  |  |  |  |  |  |  |
| 12 | 0 | 0.000999 | 0.050695 | 1 | 40.14286 | 8.571429 |
| 19 | 0 | 0.000999 | 0.050695 | 1 | 945.5714 | 3768.571 |
| 20 | 0 | 0.000999 | 0.050695 | 1 | 15015.57 | 3756.857 |
| 73 | 0 | 0.000999 | 0.050695 | 1 | 7 | 0.142857 |
| 188 | 1 | 0.000999 | 0.050695 | 1 | 20.42857 | 319.8571 |
| 196 | 0 | 0.000999 | 0.050695 | 1 | 45.14286 | 0.571429 |
| 197 | 0 | 0.000999 | 0.050695 | 1 | 35 | 0.428571 |
| 213 | 0 | 0.000999 | 0.050695 | 1 | 135.5714 | 3.571429 |
| 337 | 0 | 0.000999 | 0.050695 | 1 | 3.428571 | 60.42857 |
| 371 | 0 | 0.000999 | 0.050695 | 1 | 11.14286 | 316.1429 |
| 412 | 0 | 0.000999 | 0.050695 | 1 | 1.714286 | 38.28571 |
| 413 | 0 | 0.000999 | 0.050695 | 1 | 249.1429 | 2161.714 |
| 444 | 3.5 | 0.000999 | 0.050695 | 1 | 2.857143 | 0 |
| 453 | 1.5 | 0.000999 | 0.050695 | 1 | 30 | 140.8571 |
| 538 | 2.5 | 0.000999 | 0.050695 | 1 | 33.14286 | 0.714286 |
| 573 | 4.5 | 0.000999 | 0.050695 | 1 | 7.285714 | 0.142857 |
| 608 | 2 | 0.000999 | 0.050695 | 1 | 38.57143 | 2.714286 |
| 659 | 0.5 | 0.000999 | 0.050695 | 1 | 0.285714 | 9 |
| 739 | 3.5 | 0.000999 | 0.050695 | 1 | 0.428571 | 3.857143 |
| 806 | 1 | 0.000999 | 0.050695 | 1 | 0.285714 | 8.428571 |
| 815 | 1.5 | 0.000999 | 0.050695 | 1 | 1.857143 | 97.85714 |
| 824 | 0 | 0.000999 | 0.050695 | 1 | 14.71429 | 1 |
| 844 | 0 | 0.000999 | 0.050695 | 1 | 2.714286 | 41.14286 |
| 856 | 0 | 0.000999 | 0.050695 | 1 | 9.428571 | 34.14286 |
| 872 | 0 | 0.000999 | 0.050695 | 1 | 1.285714 | 12.28571 |
| 892 | 0.5 | 0.000999 | 0.050695 | 1 | 15.71429 | 0.714286 |
| 1211 | 1 | 0.000999 | 0.050695 | 1 | 0.285714 | 13.42857 |
| 1256 | 1.5 | 0.000999 | 0.050695 | 1 | 0.857143 | 186.7143 |
| 1334 | 4 | 0.000999 | 0.050695 | 1 | 3.285714 | 15.85714 |
| 1380 | 0 | 0.000999 | 0.050695 | 1 | 14.28571 | 0.142857 |
| 1416 | 2 | 0.000999 | 0.050695 | 1 | 12 | 1.714286 |
| 1430 | 0 | 0.000999 | 0.050695 | 1 | 0.142857 | 30.14286 |
| 1432 | 0 | 0.000999 | 0.050695 | 1 | 11.28571 | 70.14286 |
| 1584 | 0 | 0.000999 | 0.050695 | 1 | 3.142857 | 16 |
| 1636 | 3 | 0.000999 | 0.050695 | 1 | 1.428571 | 18.42857 |
| 1656 | 3 | 0.000999 | 0.050695 | 1 | 1.285714 | 12.28571 |
| 1767 | 0 | 0.000999 | 0.050695 | 1 | 10.57143 | 76 |
| 1842 | 1 | 0.000999 | 0.050695 | 1 | 16 | 2.285714 |
| 1853 | 3.5 | 0.000999 | 0.050695 | 1 | 2.285714 | 0 |
| 1870 | 0 | 0.000999 | 0.050695 | 1 | 1.714286 | 13.14286 |
| 1892 | 1 | 0.000999 | 0.050695 | 1 | 0.857143 | 15.14286 |
| 1914 | 1 | 0.000999 | 0.050695 | 1 | 1.714286 | 15 |
| 2021 | 1 | 0.000999 | 0.050695 | 1 | 2.285714 | 14.14286 |
| 2041 | 1.5 | 0.000999 | 0.050695 | 1 | 1 | 4.285714 |
| 2043 | 1.5 | 0.000999 | 0.050695 | 1 | 0.142857 | 3.571429 |
| 2149 | 2.5 | 0.000999 | 0.050695 | 1 | 1.428571 | 5.571429 |
| 2194 | 2 | 0.000999 | 0.050695 | 1 | 1.571429 | 12.57143 |
| 2210 | 0.5 | 0.000999 | 0.050695 | 1 | 15.57143 | 0.285714 |
| 2216 | 1 | 0.000999 | 0.050695 | 1 | 21.85714 | 0.714286 |
| 2222 | 1.5 | 0.000999 | 0.050695 | 1 | 8.857143 | 0.428571 |
| 2223 | 1 | 0.000999 | 0.050695 | 1 | 0.285714 | 50.71429 |
| 2227 | 4.5 | 0.000999 | 0.050695 | 1 | 0.142857 | 12.57143 |
| 2259 | 0 | 0.000999 | 0.050695 | 1 | 3.714286 | 53.71429 |
| 2265 | 0 | 0.000999 | 0.050695 | 1 | 0.285714 | 10.42857 |
| 2269 | 4.5 | 0.000999 | 0.050695 | 1 | 0.142857 | 3.571429 |
| 2279 | 1 | 0.000999 | 0.050695 | 1 | 1.142857 | 18 |
| 2283 | 1 | 0.000999 | 0.050695 | 1 | 9.857143 | 0.285714 |
| 2293 | 2 | 0.000999 | 0.050695 | 1 | 0.857143 | 4.571429 |
| 2301 | 2 | 0.000999 | 0.050695 | 1 | 1.571429 | 5.571429 |
| 2304 | 3.5 | 0.000999 | 0.050695 | 1 | 3.571429 | 0 |
| 2307 | 0.5 | 0.000999 | 0.050695 | 1 | 0.428571 | 12.85714 |
| 2314 | 0 | 0.000999 | 0.050695 | 1 | 2.142857 | 54.85714 |
| 2334 | 0.5 | 0.000999 | 0.050695 | 1 | 0.857143 | 22.14286 |
| 2397 | 0.5 | 0.000999 | 0.050695 | 1 | 3.142857 | 0.142857 |
| 2404 | 0 | 0.000999 | 0.050695 | 1 | 0.428571 | 5.857143 |
| 2477 | 2 | 0.000999 | 0.050695 | 1 | 3.142857 | 17.42857 |
| 2483 | 1 | 0.000999 | 0.050695 | 1 | 7.857143 | 0.285714 |
| 2494 | 1.5 | 0.000999 | 0.050695 | 1 | 4 | 13.14286 |
| 2495 | 0 | 0.000999 | 0.050695 | 1 | 0.285714 | 9.285714 |
| 2526 | 0 | 0.000999 | 0.050695 | 1 | 3.142857 | 37.28571 |
| 2545 | 0.5 | 0.000999 | 0.050695 | 1 | 0.428571 | 11.14286 |
| 2586 | 0 | 0.000999 | 0.050695 | 1 | 3.428571 | 57.71429 |
| 2613 | 3.5 | 0.000999 | 0.050695 | 1 | 0 | 2.142857 |
| 2668 | 3.5 | 0.000999 | 0.050695 | 1 | 0 | 5.571429 |
| 2795 | 0.5 | 0.000999 | 0.050695 | 1 | 0.142857 | 14 |
| 3014 | 2 | 0.000999 | 0.050695 | 1 | 1.285714 | 9 |
| 3031 | 0 | 0.000999 | 0.050695 | 1 | 0 | 10.85714 |
| 3110 | 0.5 | 0.000999 | 0.050695 | 1 | 2.428571 | 18 |
| 3121 | 1 | 0.000999 | 0.050695 | 1 | 0.285714 | 3.571429 |
| 3135 | 3 | 0.000999 | 0.050695 | 1 | 0.857143 | 12.28571 |
| 3251 | 0.5 | 0.000999 | 0.050695 | 1 | 0.142857 | 4.714286 |
| 3274 | 3.5 | 0.000999 | 0.050695 | 1 | 0 | 4 |
| 3285 | 2 | 0.000999 | 0.050695 | 1 | 0.285714 | 5 |
| 3466 | 0 | 0.000999 | 0.050695 | 1 | 0.428571 | 4.428571 |
| 3651 | 2 | 0.000999 | 0.050695 | 1 | 2 | 16.57143 |
| 3671 | 0 | 0.000999 | 0.050695 | 1 | 0.285714 | 8.428571 |
| 3693 | 1.5 | 0.000999 | 0.050695 | 1 | 0.428571 | 4.571429 |
| 3787 | 3.5 | 0.000999 | 0.050695 | 1 | 0 | 11.57143 |
| 3797 | 0 | 0.000999 | 0.050695 | 1 | 9.714286 | 0.285714 |
| 3888 | 0 | 0.000999 | 0.050695 | 1 | 1.571429 | 10.85714 |
| 3948 | 0 | 0.000999 | 0.050695 | 1 | 3.714286 | 0 |
| 3985 | 0 | 0.000999 | 0.050695 | 1 | 0.142857 | 4.142857 |
| 4084 | 7 | 0.000999 | 0.050695 | 1 | 0 | 0.857143 |
| 4378 | 0 | 0.000999 | 0.050695 | 1 | 14.42857 | 0.142857 |
| 4552 | 3.5 | 0.000999 | 0.050695 | 1 | 0.857143 | 0 |
| 4609 | 0 | 0.000999 | 0.050695 | 1 | 4.285714 | 0.571429 |
| 4838 | 3 | 0.000999 | 0.050695 | 1 | 6 | 0.714286 |
| 4970 | 1 | 0.000999 | 0.050695 | 1 | 0.571429 | 4.857143 |
| 4984 | 1 | 0.000999 | 0.050695 | 1 | 23.85714 | 2.428571 |
| 5029 | 3.5 | 0.000999 | 0.050695 | 1 | 0 | 4.857143 |
| 5146 | 0 | 0.000999 | 0.050695 | 1 | 14 | 0.714286 |
| 5270 | 3.5 | 0.000999 | 0.050695 | 1 | 3.285714 | 0 |
| 5275 | 0 | 0.000999 | 0.050695 | 1 | 11.28571 | 0.142857 |
| 5416 | 0 | 0.000999 | 0.050695 | 1 | 13.85714 | 0 |
| 5505 | 0 | 0.000999 | 0.050695 | 1 | 4.857143 | 28.85714 |
| 5540 | 7 | 0.000999 | 0.050695 | 1 | 0 | 2.428571 |
| 5785 | 1 | 0.000999 | 0.050695 | 1 | 0.285714 | 6.857143 |
| 5786 | 3.5 | 0.000999 | 0.050695 | 1 | 0 | 2.285714 |
| 5952 | 2.5 | 0.000999 | 0.050695 | 1 | 0.714286 | 7.857143 |
| 6096 | 0 | 0.000999 | 0.050695 | 1 | 0 | 7.428571 |
| 6115 | 0 | 0.000999 | 0.050695 | 1 | 1.428571 | 21.42857 |
| 6192 | 2 | 0.000999 | 0.050695 | 1 | 3.142857 | 13.71429 |
| 6205 | 0 | 0.000999 | 0.050695 | 1 | 0 | 4.714286 |
| 6215 | 4 | 0.000999 | 0.050695 | 1 | 0.285714 | 5 |
| 6218 | 2.5 | 0.000999 | 0.050695 | 1 | 0.714286 | 5.285714 |
| 6291 | 3 | 0.000999 | 0.050695 | 1 | 0.285714 | 4.714286 |
| 6328 | 0 | 0.000999 | 0.050695 | 1 | 1.571429 | 147 |
| 6390 | 4.5 | 0.000999 | 0.050695 | 1 | 0.571429 | 17.14286 |
| 6491 | 0 | 0.000999 | 0.050695 | 1 | 1 | 7.571429 |
| 6577 | 3.5 | 0.000999 | 0.050695 | 1 | 0 | 2 |
| 6614 | 3.5 | 0.000999 | 0.050695 | 1 | 0 | 6.571429 |
| 7990 | 0 | 0.000999 | 0.050695 | 1 | 0 | 5.428571 |
| 8610 | 7 | 0.000999 | 0.050695 | 1 | 0.714286 | 0 |
| 9012 | 1 | 0.000999 | 0.050695 | 1 | 1.142857 | 5.142857 |
| 93 | 2.5 | 0.001998 | 0.069431 | 1 | 0.857143 | 6.285714 |
| 95 | 2 | 0.001998 | 0.069431 | 1 | 31.14286 | 81.57143 |
| 170 | 2.5 | 0.001998 | 0.069431 | 1 | 25.85714 | 2.714286 |
| 178 | 1 | 0.001998 | 0.069431 | 1 | 7.142857 | 0.142857 |
| 278 | 3 | 0.001998 | 0.069431 | 1 | 3 | 183.8571 |
| 281 | 1.5 | 0.001998 | 0.069431 | 1 | 13.85714 | 0.428571 |
| 282 | 2.5 | 0.001998 | 0.069431 | 1 | 1.571429 | 6.714286 |
| 295 | 4 | 0.001998 | 0.069431 | 1 | 11.14286 | 1.571429 |
| 457 | 2 | 0.001998 | 0.069431 | 1 | 3.142857 | 87.57143 |
| 821 | 1 | 0.001998 | 0.069431 | 1 | 0.285714 | 3.428571 |
| 837 | 4.5 | 0.001998 | 0.069431 | 1 | 5.428571 | 0.142857 |
| 858 | 7 | 0.001998 | 0.069431 | 1 | 3.714286 | 0 |
| 870 | 3.5 | 0.001998 | 0.069431 | 1 | 0.571429 | 6.428571 |
| 916 | 3 | 0.001998 | 0.069431 | 1 | 32.42857 | 1.142857 |
| 943 | 2.5 | 0.001998 | 0.069431 | 1 | 1.571429 | 7.857143 |
| 1025 | 5.5 | 0.001998 | 0.069431 | 1 | 0.142857 | 1.857143 |
| 1064 | 3.5 | 0.001998 | 0.069431 | 1 | 2 | 0 |
| 1226 | 5 | 0.001998 | 0.069431 | 1 | 3.714286 | 0.142857 |
| 1465 | 4 | 0.001998 | 0.069431 | 1 | 1 | 5.571429 |
| 1751 | 3.5 | 0.001998 | 0.069431 | 1 | 3 | 0 |
| 1840 | 3 | 0.001998 | 0.069431 | 1 | 0.428571 | 14.71429 |
| 1913 | 4.5 | 0.001998 | 0.069431 | 1 | 0.857143 | 5.571429 |
| 2014 | 2 | 0.001998 | 0.069431 | 1 | 0.428571 | 7.571429 |
| 2055 | 1 | 0.001998 | 0.069431 | 1 | 3.285714 | 17.14286 |
| 2139 | 2.5 | 0.001998 | 0.069431 | 1 | 0.857143 | 9.571429 |
| 2152 | 1 | 0.001998 | 0.069431 | 1 | 4.285714 | 0.142857 |
| 2218 | 2 | 0.001998 | 0.069431 | 1 | 3 | 11.71429 |
| 2236 | 3.5 | 0.001998 | 0.069431 | 1 | 4.285714 | 18 |
| 2450 | 4.5 | 0.001998 | 0.069431 | 1 | 2.142857 | 7 |
| 2473 | 3.5 | 0.001998 | 0.069431 | 1 | 7.571429 | 42.85714 |
| 2574 | 10.5 | 0.001998 | 0.069431 | 1 | 0 | 0.571429 |
| 2982 | 2 | 0.001998 | 0.069431 | 1 | 2 | 20.71429 |
| 2984 | 3 | 0.001998 | 0.069431 | 1 | 3.142857 | 66 |
| 3150 | 2.5 | 0.001998 | 0.069431 | 1 | 1.142857 | 10.57143 |
| 3199 | 3 | 0.001998 | 0.069431 | 1 | 4.714286 | 49.57143 |
| 3353 | 0.5 | 0.001998 | 0.069431 | 1 | 0.857143 | 18.14286 |
| 3415 | 3.5 | 0.001998 | 0.069431 | 1 | 0 | 9.571429 |
| 3808 | 5.5 | 0.001998 | 0.069431 | 1 | 0.142857 | 1.857143 |
| 4030 | 3.5 | 0.001998 | 0.069431 | 1 | 0 | 1.571429 |
| 4115 | 4 | 0.001998 | 0.069431 | 1 | 0.857143 | 5.285714 |
| 4401 | 7 | 0.001998 | 0.069431 | 1 | 2 | 0 |
| 4740 | 5 | 0.001998 | 0.069431 | 1 | 2 | 0.142857 |
| 4855 | 3.5 | 0.001998 | 0.069431 | 1 | 1.285714 | 0 |
| 4954 | 1 | 0.001998 | 0.069431 | 1 | 0.714286 | 7.285714 |
| 5055 | 4 | 0.001998 | 0.069431 | 1 | 0.285714 | 3.714286 |
| 5465 | 1.5 | 0.001998 | 0.069431 | 1 | 18.28571 | 2 |
| 5763 | 3 | 0.001998 | 0.069431 | 1 | 0.428571 | 3.428571 |
| 5766 | 3.5 | 0.001998 | 0.069431 | 1 | 0 | 2.285714 |
| 5975 | 3.5 | 0.001998 | 0.069431 | 1 | 0 | 2.428571 |
| 6111 | 1 | 0.001998 | 0.069431 | 1 | 4 | 21.71429 |
| 6306 | 3.5 | 0.001998 | 0.069431 | 1 | 1.142857 | 7.857143 |
| 6445 | 7 | 0.001998 | 0.069431 | 1 | 0 | 3.285714 |
| 6471 | 3 | 0.001998 | 0.069431 | 1 | 0.285714 | 8.285714 |
| 6622 | 1 | 0.001998 | 0.069431 | 1 | 1 | 20.14286 |
| 6682 | 7 | 0.001998 | 0.069431 | 1 | 0 | 0.714286 |
| 6852 | 3.5 | 0.001998 | 0.069431 | 1 | 0 | 2.142857 |
| 7406 | 3.5 | 0.001998 | 0.069431 | 1 | 0 | 1.857143 |
| 8528 | 4 | 0.001998 | 0.069431 | 1 | 7.857143 | 0.142857 |
| 230 | 4.5 | 0.002997 | 0.084048 | 1 | 7.428571 | 0.142857 |
| 248 | 1 | 0.002997 | 0.084048 | 1 | 27.71429 | 181.4286 |
| 385 | 7 | 0.002997 | 0.084048 | 1 | 10.28571 | 0 |
| 579 | 2.5 | 0.002997 | 0.084048 | 1 | 2.571429 | 43.28571 |
| 589 | 4 | 0.002997 | 0.084048 | 1 | 1.285714 | 12.85714 |
| 663 | 7 | 0.002997 | 0.084048 | 1 | 1.285714 | 0 |
| 833 | 2.5 | 0.002997 | 0.084048 | 1 | 40 | 1.428571 |
| 1214 | 3.5 | 0.002997 | 0.084048 | 1 | 3.142857 | 38.14286 |
| 1317 | 3 | 0.002997 | 0.084048 | 1 | 0.857143 | 128.5714 |
| 1583 | 7 | 0.002997 | 0.084048 | 1 | 7.142857 | 0 |
| 1639 | 4 | 0.002997 | 0.084048 | 1 | 5.428571 | 37.42857 |
| 1787 | 2.5 | 0.002997 | 0.084048 | 1 | 0.857143 | 32.42857 |
| 1829 | 3 | 0.002997 | 0.084048 | 1 | 3.857143 | 19.71429 |
| 1907 | 2.5 | 0.002997 | 0.084048 | 1 | 5.571429 | 49.85714 |
| 2027 | 3.5 | 0.002997 | 0.084048 | 1 | 3.285714 | 0 |
| 2195 | 4 | 0.002997 | 0.084048 | 1 | 2 | 13.57143 |
| 2208 | 5.5 | 0.002997 | 0.084048 | 1 | 4 | 0.285714 |
| 2260 | 3 | 0.002997 | 0.084048 | 1 | 1.142857 | 10.57143 |
| 2267 | 5.5 | 0.002997 | 0.084048 | 1 | 3.285714 | 0.142857 |
| 2276 | 3 | 0.002997 | 0.084048 | 1 | 5.857143 | 27.14286 |
| 2338 | 2 | 0.002997 | 0.084048 | 1 | 0.571429 | 7.285714 |
| 2429 | 3.5 | 0.002997 | 0.084048 | 1 | 10.14286 | 1 |
| 2557 | 7 | 0.002997 | 0.084048 | 1 | 0.857143 | 0 |
| 2730 | 4 | 0.002997 | 0.084048 | 1 | 1.142857 | 7.285714 |
| 2993 | 7 | 0.002997 | 0.084048 | 1 | 0 | 5 |
| 3577 | 4 | 0.002997 | 0.084048 | 1 | 0.285714 | 1.857143 |
| 3599 | 7 | 0.002997 | 0.084048 | 1 | 0 | 2.142857 |
| 3742 | 3.5 | 0.002997 | 0.084048 | 1 | 8.428571 | 0 |
| 3744 | 4.5 | 0.002997 | 0.084048 | 1 | 1 | 5.857143 |
| 3838 | 2.5 | 0.002997 | 0.084048 | 1 | 10.42857 | 1 |
| 3944 | 5.5 | 0.002997 | 0.084048 | 1 | 0.428571 | 3.142857 |
| 4351 | 7 | 0.002997 | 0.084048 | 1 | 1 | 0 |
| 4424 | 5.5 | 0.002997 | 0.084048 | 1 | 0.428571 | 5.714286 |
| 4734 | 4.5 | 0.002997 | 0.084048 | 1 | 5.285714 | 0.285714 |
| 5253 | 3.5 | 0.002997 | 0.084048 | 1 | 0 | 1.714286 |
| 5778 | 3.5 | 0.002997 | 0.084048 | 1 | 0.714286 | 7.142857 |
| 5973 | 7 | 0.002997 | 0.084048 | 1 | 0 | 1.142857 |
| 6031 | 1 | 0.002997 | 0.084048 | 1 | 1.142857 | 7.571429 |
| 6342 | 1 | 0.002997 | 0.084048 | 1 | 0.285714 | 10.42857 |
| 6646 | 4.5 | 0.002997 | 0.084048 | 1 | 0.142857 | 4.714286 |
| 7069 | 7 | 0.002997 | 0.084048 | 1 | 0 | 2 |
| 7122 | 4 | 0.002997 | 0.084048 | 1 | 0.142857 | 13.28571 |
| 7139 | 7 | 0.002997 | 0.084048 | 1 | 0 | 2.142857 |
| 8013 | 2 | 0.002997 | 0.084048 | 1 | 2.857143 | 8.285714 |
| 37 | 1 | 0.003996 | 0.094983 | 1 | 1347.286 | 153.7143 |
| 44 | 2 | 0.003996 | 0.094983 | 1 | 155.1429 | 16.85714 |
| 148 | 3 | 0.003996 | 0.094983 | 1 | 95 | 713.7143 |
| 155 | 5 | 0.003996 | 0.094983 | 1 | 4.428571 | 20.42857 |
| 354 | 3.5 | 0.003996 | 0.094983 | 1 | 39.28571 | 5.571429 |
| 849 | 7 | 0.003996 | 0.094983 | 1 | 0 | 0.857143 |
| 903 | 3 | 0.003996 | 0.094983 | 1 | 12.14286 | 0.428571 |
| 904 | 7 | 0.003996 | 0.094983 | 1 | 7 | 0 |
| 1389 | 5.5 | 0.003996 | 0.094983 | 1 | 0.285714 | 8.142857 |
| 1393 | 5.5 | 0.003996 | 0.094983 | 1 | 5.571429 | 0.142857 |
| 1565 | 3 | 0.003996 | 0.094983 | 1 | 2.285714 | 53.57143 |
| 1833 | 4.5 | 0.003996 | 0.094983 | 1 | 3.285714 | 0.428571 |
| 1931 | 3 | 0.003996 | 0.094983 | 1 | 17.42857 | 2.142857 |
| 1950 | 4.5 | 0.003996 | 0.094983 | 1 | 1.857143 | 38.14286 |
| 2241 | 2 | 0.003996 | 0.094983 | 1 | 2.428571 | 15 |
| 2296 | 4 | 0.003996 | 0.094983 | 1 | 1.428571 | 12.71429 |
| 2373 | 7 | 0.003996 | 0.094983 | 1 | 2.857143 | 0 |
| 2499 | 4.5 | 0.003996 | 0.094983 | 1 | 0.428571 | 6.857143 |
| 2524 | 3.5 | 0.003996 | 0.094983 | 1 | 1 | 12.85714 |
| 2542 | 6 | 0.003996 | 0.094983 | 1 | 4.142857 | 1.714286 |
| 3134 | 4 | 0.003996 | 0.094983 | 1 | 0.142857 | 13.28571 |
| 3237 | 4.5 | 0.003996 | 0.094983 | 1 | 1.428571 | 7.857143 |
| 3335 | 2 | 0.003996 | 0.094983 | 1 | 0.571429 | 6.428571 |
| 3394 | 4.5 | 0.003996 | 0.094983 | 1 | 1.142857 | 19 |
| 3529 | 7 | 0.003996 | 0.094983 | 1 | 0 | 4 |
| 3690 | 4 | 0.003996 | 0.094983 | 1 | 2.428571 | 25.71429 |
| 3880 | 3 | 0.003996 | 0.094983 | 1 | 0.714286 | 7.857143 |
| 4121 | 5 | 0.003996 | 0.094983 | 1 | 0.714286 | 15.71429 |
| 4960 | 3.5 | 0.003996 | 0.094983 | 1 | 1.142857 | 8.285714 |
| 5078 | 1.5 | 0.003996 | 0.094983 | 1 | 1.857143 | 17 |
| 5739 | 10.5 | 0.003996 | 0.094983 | 1 | 0 | 0.571429 |
| 6124 | 7 | 0.003996 | 0.094983 | 1 | 0 | 2.285714 |
| 6305 | 5 | 0.003996 | 0.094983 | 1 | 0.428571 | 5.428571 |
| 6343 | 2.5 | 0.003996 | 0.094983 | 1 | 1.857143 | 7.714286 |
| 6631 | 7 | 0.003996 | 0.094983 | 1 | 0 | 1.142857 |
| 6776 | 3.5 | 0.003996 | 0.094983 | 1 | 2.714286 | 9.428571 |
| 6854 | 10.5 | 0.003996 | 0.094983 | 1 | 0 | 0.571429 |
| 6961 | 7 | 0.003996 | 0.094983 | 1 | 0 | 2 |
| 7081 | 6.5 | 0.003996 | 0.094983 | 1 | 0.285714 | 1.571429 |
| 7810 | 7 | 0.003996 | 0.094983 | 1 | 3.142857 | 0 |
| 9131 | 7 | 0.003996 | 0.094983 | 1 | 1.285714 | 0 |
| 27 | 5 | 0.004995 | 0.102695 | 1 | 420.1429 | 147.8571 |
| 48 | 4.5 | 0.004995 | 0.102695 | 1 | 5.285714 | 189.7143 |
| 78 | 7 | 0.004995 | 0.102695 | 1 | 2.571429 | 0 |
| 130 | 2.5 | 0.004995 | 0.102695 | 1 | 2.571429 | 13.85714 |
| 209 | 4 | 0.004995 | 0.102695 | 1 | 3.714286 | 51.28571 |
| 403 | 5.5 | 0.004995 | 0.102695 | 1 | 3.142857 | 0.285714 |
| 475 | 3 | 0.004995 | 0.102695 | 1 | 40.57143 | 6.571429 |
| 483 | 5 | 0.004995 | 0.102695 | 1 | 14.71429 | 67.42857 |
| 510 | 4.5 | 0.004995 | 0.102695 | 1 | 12.57143 | 0.142857 |
| 802 | 4.5 | 0.004995 | 0.102695 | 1 | 29.42857 | 0.571429 |
| 1143 | 5 | 0.004995 | 0.102695 | 1 | 3.571429 | 0.142857 |
| 1175 | 7 | 0.004995 | 0.102695 | 1 | 1.142857 | 0 |
| 1444 | 6 | 0.004995 | 0.102695 | 1 | 0.714286 | 16 |
| 1668 | 4 | 0.004995 | 0.102695 | 1 | 3.714286 | 19.57143 |
| 1865 | 6.5 | 0.004995 | 0.102695 | 1 | 0.285714 | 3.285714 |
| 1880 | 2.5 | 0.004995 | 0.102695 | 1 | 3.571429 | 15.42857 |
| 2263 | 4 | 0.004995 | 0.102695 | 1 | 19.71429 | 2.142857 |
| 2277 | 5 | 0.004995 | 0.102695 | 1 | 1.857143 | 0.142857 |
| 2406 | 5.5 | 0.004995 | 0.102695 | 1 | 2.714286 | 0.142857 |
| 2445 | 4 | 0.004995 | 0.102695 | 1 | 5.142857 | 19.28571 |
| 2476 | 5 | 0.004995 | 0.102695 | 1 | 0.142857 | 5.142857 |
| 2806 | 3 | 0.004995 | 0.102695 | 1 | 2.714286 | 49.71429 |
| 2996 | 7 | 0.004995 | 0.102695 | 1 | 1.142857 | 0 |
| 3007 | 5.5 | 0.004995 | 0.102695 | 1 | 0.285714 | 3.714286 |
| 3193 | 7 | 0.004995 | 0.102695 | 1 | 2.714286 | 0 |
| 3217 | 7 | 0.004995 | 0.102695 | 1 | 0 | 25 |
| 3268 | 5.5 | 0.004995 | 0.102695 | 1 | 0.285714 | 2.571429 |
| 4008 | 4.5 | 0.004995 | 0.102695 | 1 | 4 | 35.28571 |
| 4215 | 10.5 | 0.004995 | 0.102695 | 1 | 0.571429 | 0 |
| 4258 | 4.5 | 0.004995 | 0.102695 | 1 | 3.857143 | 0.571429 |
| 4686 | 4.5 | 0.004995 | 0.102695 | 1 | 5.857143 | 0.142857 |
| 4737 | 7 | 0.004995 | 0.102695 | 1 | 1.142857 | 0 |
| 4763 | 7 | 0.004995 | 0.102695 | 1 | 3.142857 | 0 |
| 5376 | 3.5 | 0.004995 | 0.102695 | 1 | 0.571429 | 2.285714 |
| 5958 | 5.5 | 0.004995 | 0.102695 | 1 | 0.285714 | 30.42857 |
| 6043 | 7 | 0.004995 | 0.102695 | 1 | 0 | 1.142857 |
| 6273 | 7 | 0.004995 | 0.102695 | 1 | 0 | 1.714286 |
| 6356 | 7 | 0.004995 | 0.102695 | 1 | 0 | 1.571429 |
| 6361 | 7 | 0.004995 | 0.102695 | 1 | 0 | 2.714286 |
| 6813 | 7 | 0.004995 | 0.102695 | 1 | 0 | 1.142857 |
| 7277 | 10.5 | 0.004995 | 0.102695 | 1 | 0 | 0.571429 |
| 8113 | 4 | 0.004995 | 0.102695 | 1 | 13.71429 | 68.85714 |
| 5 | 5 | 0.005994 | 0.107055 | 1 | 197.8571 | 543.1429 |
| 8 | 5 | 0.005994 | 0.107055 | 1 | 2773.429 | 776.4286 |
| 13 | 5 | 0.005994 | 0.107055 | 1 | 20.28571 | 2404.429 |
| 71 | 5 | 0.005994 | 0.107055 | 1 | 224.4286 | 2135 |
| 194 | 4.5 | 0.005994 | 0.107055 | 1 | 10 | 0.285714 |
| 416 | 7 | 0.005994 | 0.107055 | 1 | 1.714286 | 0 |
| 934 | 5.5 | 0.005994 | 0.107055 | 1 | 5.285714 | 22.14286 |
| 975 | 5.5 | 0.005994 | 0.107055 | 1 | 1.714286 | 22.85714 |
| 1017 | 4 | 0.005994 | 0.107055 | 1 | 6.857143 | 49.71429 |
| 1110 | 5 | 0.005994 | 0.107055 | 1 | 0.285714 | 5.285714 |
| 1197 | 5.5 | 0.005994 | 0.107055 | 1 | 0.714286 | 6.428571 |
| 1512 | 4 | 0.005994 | 0.107055 | 1 | 1.428571 | 22.57143 |
| 1532 | 5.5 | 0.005994 | 0.107055 | 1 | 3 | 29.42857 |
| 1572 | 3.5 | 0.005994 | 0.107055 | 1 | 0.857143 | 6.571429 |
| 1591 | 4.5 | 0.005994 | 0.107055 | 1 | 5.714286 | 0.571429 |
| 1835 | 5.5 | 0.005994 | 0.107055 | 1 | 0.285714 | 5 |
| 1928 | 2 | 0.005994 | 0.107055 | 1 | 9.285714 | 52.42857 |
| 1974 | 7 | 0.005994 | 0.107055 | 1 | 13.57143 | 0 |
| 2199 | 6 | 0.005994 | 0.107055 | 1 | 0.571429 | 7.857143 |
| 2858 | 3.5 | 0.005994 | 0.107055 | 1 | 0.714286 | 7.857143 |
| 2953 | 5 | 0.005994 | 0.107055 | 1 | 0.857143 | 7.428571 |
| 3000 | 3.5 | 0.005994 | 0.107055 | 1 | 0.857143 | 7.285714 |
| 3079 | 3.5 | 0.005994 | 0.107055 | 1 | 2.428571 | 13.28571 |
| 3333 | 7 | 0.005994 | 0.107055 | 1 | 1.285714 | 0 |
| 3369 | 6 | 0.005994 | 0.107055 | 1 | 0.714286 | 7.714286 |
| 3679 | 5 | 0.005994 | 0.107055 | 1 | 0.571429 | 14.71429 |
| 3704 | 5.5 | 0.005994 | 0.107055 | 1 | 0.285714 | 3.714286 |
| 3960 | 10.5 | 0.005994 | 0.107055 | 1 | 0.571429 | 0 |
| 3962 | 3 | 0.005994 | 0.107055 | 1 | 6.142857 | 31 |
| 4007 | 4.5 | 0.005994 | 0.107055 | 1 | 0.285714 | 8.714286 |
| 4305 | 5.5 | 0.005994 | 0.107055 | 1 | 0.571429 | 6.857143 |
| 4331 | 5.5 | 0.005994 | 0.107055 | 1 | 0.857143 | 11.28571 |
| 4529 | 10.5 | 0.005994 | 0.107055 | 1 | 1.142857 | 0 |
| 4533 | 9.5 | 0.005994 | 0.107055 | 1 | 0.142857 | 1.142857 |
| 4790 | 7 | 0.005994 | 0.107055 | 1 | 1.428571 | 0 |
| 5018 | 6 | 0.005994 | 0.107055 | 1 | 0.428571 | 3.571429 |
| 5083 | 6.5 | 0.005994 | 0.107055 | 1 | 0.571429 | 1.714286 |
| 6313 | 5.5 | 0.005994 | 0.107055 | 1 | 0.285714 | 2.285714 |
| 6603 | 7 | 0.005994 | 0.107055 | 1 | 0 | 0.857143 |
| 6812 | 4.5 | 0.005994 | 0.107055 | 1 | 1.142857 | 15.28571 |
| 7215 | 10.5 | 0.005994 | 0.107055 | 1 | 0 | 0.571429 |
| 7800 | 7 | 0.005994 | 0.107055 | 1 | 1.714286 | 0 |
| 7947 | 7 | 0.005994 | 0.107055 | 1 | 1.857143 | 0 |
| 7948 | 5 | 0.005994 | 0.107055 | 1 | 0.142857 | 5.571429 |
| 8435 | 10.5 | 0.005994 | 0.107055 | 1 | 0 | 0.571429 |
| 8461 | 10.5 | 0.005994 | 0.107055 | 1 | 0 | 2.857143 |
| 8656 | 10.5 | 0.005994 | 0.107055 | 1 | 0.571429 | 0 |
| 141 | 4 | 0.006993 | 0.116745 | 1 | 10.85714 | 484.7143 |
| 360 | 3.5 | 0.006993 | 0.116745 | 1 | 1.428571 | 12.57143 |
| 1217 | 5 | 0.006993 | 0.116745 | 1 | 3.571429 | 29.85714 |
| 1582 | 6 | 0.006993 | 0.116745 | 1 | 0.142857 | 1.142857 |
| 1711 | 7 | 0.006993 | 0.116745 | 1 | 0.571429 | 11.14286 |
| 1831 | 6 | 0.006993 | 0.116745 | 1 | 8.714286 | 0.285714 |
| 2219 | 4.5 | 0.006993 | 0.116745 | 1 | 15 | 45.85714 |
| 2536 | 5.5 | 0.006993 | 0.116745 | 1 | 7 | 1.857143 |
| 3272 | 5.5 | 0.006993 | 0.116745 | 1 | 1 | 8.428571 |
| 3364 | 6 | 0.006993 | 0.116745 | 1 | 0.714286 | 6.857143 |
| 3776 | 4.5 | 0.006993 | 0.116745 | 1 | 5.571429 | 20.28571 |
| 3899 | 5 | 0.006993 | 0.116745 | 1 | 0.428571 | 3.571429 |
| 4802 | 10.5 | 0.006993 | 0.116745 | 1 | 0.571429 | 0 |
| 4890 | 10.5 | 0.006993 | 0.116745 | 1 | 0.571429 | 0 |
| 4933 | 5 | 0.006993 | 0.116745 | 1 | 3.285714 | 0.142857 |
| 5024 | 5 | 0.006993 | 0.116745 | 1 | 0.428571 | 6.571429 |
| 5471 | 6.5 | 0.006993 | 0.116745 | 1 | 0.285714 | 2.142857 |
| 6046 | 7 | 0.006993 | 0.116745 | 1 | 0 | 2 |
| 6392 | 6.5 | 0.006993 | 0.116745 | 1 | 0.285714 | 3 |
| 6700 | 6 | 0.006993 | 0.116745 | 1 | 0.285714 | 2.857143 |
| 6794 | 5.5 | 0.006993 | 0.116745 | 1 | 0.142857 | 2.285714 |
| 6797 | 10.5 | 0.006993 | 0.116745 | 1 | 0 | 1 |
| 7907 | 10.5 | 0.006993 | 0.116745 | 1 | 1.857143 | 0 |
| 7958 | 5 | 0.006993 | 0.116745 | 1 | 9.714286 | 0.428571 |
| 8128 | 4.5 | 0.006993 | 0.116745 | 1 | 10.57143 | 1.285714 |
| 210 | 6 | 0.007992 | 0.124941 | 1 | 22.42857 | 129.7143 |
| 929 | 5.5 | 0.007992 | 0.124941 | 1 | 17.42857 | 0.857143 |
| 1004 | 6 | 0.007992 | 0.124941 | 1 | 7.571429 | 0.857143 |
| 1052 | 4.5 | 0.007992 | 0.124941 | 1 | 0.142857 | 7.857143 |
| 1183 | 8 | 0.007992 | 0.124941 | 1 | 0.142857 | 5 |
| 1300 | 6 | 0.007992 | 0.124941 | 1 | 2.285714 | 0.142857 |
| 1682 | 5 | 0.007992 | 0.124941 | 1 | 2.285714 | 8.285714 |
| 1813 | 10.5 | 0.007992 | 0.124941 | 1 | 0 | 0.857143 |
| 1830 | 5 | 0.007992 | 0.124941 | 1 | 0.285714 | 4.857143 |
| 1903 | 4.5 | 0.007992 | 0.124941 | 1 | 13.14286 | 0.428571 |
| 1954 | 6.5 | 0.007992 | 0.124941 | 1 | 3.714286 | 31.71429 |
| 2618 | 10.5 | 0.007992 | 0.124941 | 1 | 0.571429 | 0 |
| 3231 | 5 | 0.007992 | 0.124941 | 1 | 1 | 8 |
| 3324 | 4.5 | 0.007992 | 0.124941 | 1 | 10.85714 | 0.285714 |
| 3438 | 6 | 0.007992 | 0.124941 | 1 | 3 | 0.428571 |
| 3478 | 7 | 0.007992 | 0.124941 | 1 | 0 | 0.857143 |
| 3825 | 7 | 0.007992 | 0.124941 | 1 | 0 | 2.714286 |
| 4077 | 5.5 | 0.007992 | 0.124941 | 1 | 0.857143 | 5.857143 |
| 4739 | 8 | 0.007992 | 0.124941 | 1 | 5.857143 | 0.142857 |
| 4963 | 10.5 | 0.007992 | 0.124941 | 1 | 0.571429 | 0 |
| 5979 | 6 | 0.007992 | 0.124941 | 1 | 1.428571 | 5 |
| 6056 | 7 | 0.007992 | 0.124941 | 1 | 0 | 1.571429 |
| 6077 | 4.5 | 0.007992 | 0.124941 | 1 | 0.285714 | 4.428571 |
| 6474 | 6 | 0.007992 | 0.124941 | 1 | 0.714286 | 4.142857 |
| 6521 | 5.5 | 0.007992 | 0.124941 | 1 | 0.285714 | 4.571429 |
| 8156 | 4 | 0.007992 | 0.124941 | 1 | 1.142857 | 6.857143 |
| 576 | 5.5 | 0.008991 | 0.133384 | 1 | 34.85714 | 6.571429 |
| 587 | 5.5 | 0.008991 | 0.133384 | 1 | 0.714286 | 8.714286 |
| 661 | 7 | 0.008991 | 0.133384 | 1 | 0 | 1.428571 |
| 767 | 5.5 | 0.008991 | 0.133384 | 1 | 2 | 7.714286 |
| 1141 | 6 | 0.008991 | 0.133384 | 1 | 0.857143 | 6.857143 |
| 1238 | 5.5 | 0.008991 | 0.133384 | 1 | 5 | 0.857143 |
| 1335 | 6 | 0.008991 | 0.133384 | 1 | 0.857143 | 4 |
| 1881 | 5 | 0.008991 | 0.133384 | 1 | 0.428571 | 7.571429 |
| 1988 | 5.5 | 0.008991 | 0.133384 | 1 | 5.571429 | 0.285714 |
| 2045 | 5.5 | 0.008991 | 0.133384 | 1 | 20.42857 | 2.428571 |
| 2114 | 5 | 0.008991 | 0.133384 | 1 | 9.285714 | 0.857143 |
| 2846 | 6 | 0.008991 | 0.133384 | 1 | 1.142857 | 8.428571 |
| 3261 | 6.5 | 0.008991 | 0.133384 | 1 | 0.142857 | 1 |
| 3891 | 5.5 | 0.008991 | 0.133384 | 1 | 3.714286 | 0.571429 |
| 3937 | 6 | 0.008991 | 0.133384 | 1 | 2.428571 | 11.57143 |
| 3941 | 6 | 0.008991 | 0.133384 | 1 | 0.428571 | 3.142857 |
| 5333 | 6.5 | 0.008991 | 0.133384 | 1 | 7.428571 | 0.571429 |
| 5383 | 5.5 | 0.008991 | 0.133384 | 1 | 0.571429 | 6.571429 |
| 5599 | 6 | 0.008991 | 0.133384 | 1 | 4.714286 | 0.428571 |
| 6542 | 10.5 | 0.008991 | 0.133384 | 1 | 0 | 1.285714 |
| 6939 | 7 | 0.008991 | 0.133384 | 1 | 0 | 2.714286 |
| 7909 | 10.5 | 0.008991 | 0.133384 | 1 | 0 | 0.857143 |
| 203 | 5.5 | 0.00999 | 0.140696 | 1 | 3 | 0.285714 |
| 582 | 8 | 0.00999 | 0.140696 | 1 | 0.142857 | 9.285714 |
| 1126 | 10.5 | 0.00999 | 0.140696 | 1 | 0 | 1.142857 |
| 1273 | 5 | 0.00999 | 0.140696 | 1 | 3.857143 | 18 |
| 1514 | 6 | 0.00999 | 0.140696 | 1 | 15.42857 | 1.857143 |
| 1741 | 10.5 | 0.00999 | 0.140696 | 1 | 0.857143 | 0 |
| 2743 | 10.5 | 0.00999 | 0.140696 | 1 | 0 | 2.571429 |
| 2814 | 10.5 | 0.00999 | 0.140696 | 1 | 0 | 0.857143 |
| 3115 | 10.5 | 0.00999 | 0.140696 | 1 | 0 | 2.428571 |
| 3758 | 7.5 | 0.00999 | 0.140696 | 1 | 0.285714 | 3.428571 |
| 3790 | 7.5 | 0.00999 | 0.140696 | 1 | 0.428571 | 4 |
| 3850 | 10.5 | 0.00999 | 0.140696 | 1 | 2.142857 | 0 |
| 4818 | 7 | 0.00999 | 0.140696 | 1 | 0.285714 | 1.857143 |
| 4887 | 8.5 | 0.00999 | 0.140696 | 1 | 4 | 0.142857 |
| 4981 | 10.5 | 0.00999 | 0.140696 | 1 | 0 | 0.571429 |
| 4987 | 6.5 | 0.00999 | 0.140696 | 1 | 0.428571 | 2.857143 |
| 5276 | 5 | 0.00999 | 0.140696 | 1 | 2.285714 | 6.714286 |
| 5468 | 10.5 | 0.00999 | 0.140696 | 1 | 1.714286 | 0 |
| 5569 | 5.5 | 0.00999 | 0.140696 | 1 | 0.285714 | 5.428571 |
| 5922 | 10.5 | 0.00999 | 0.140696 | 1 | 0.571429 | 0 |
| 6069 | 10.5 | 0.00999 | 0.140696 | 1 | 0 | 0.714286 |
| 6193 | 7 | 0.00999 | 0.140696 | 1 | 0 | 1.714286 |
| 6617 | 5.5 | 0.00999 | 0.140696 | 1 | 0.285714 | 4.857143 |
| 392 | 10.5 | 0.010989 | 0.148236 | 1 | 1.285714 | 0 |
| 537 | 5 | 0.010989 | 0.148236 | 1 | 36.14286 | 11.42857 |
| 637 | 10.5 | 0.010989 | 0.148236 | 1 | 0 | 1.142857 |
| 645 | 10.5 | 0.010989 | 0.148236 | 1 | 0 | 2.428571 |
| 689 | 8.5 | 0.010989 | 0.148236 | 1 | 8.142857 | 0.428571 |
| 871 | 10.5 | 0.010989 | 0.148236 | 1 | 6 | 0 |
| 979 | 5.5 | 0.010989 | 0.148236 | 1 | 2 | 10.42857 |
| 1051 | 6.5 | 0.010989 | 0.148236 | 1 | 1.142857 | 6.142857 |
| 1258 | 7 | 0.010989 | 0.148236 | 1 | 1.285714 | 15.14286 |
| 2412 | 5.5 | 0.010989 | 0.148236 | 1 | 7.285714 | 43.57143 |
| 2437 | 10.5 | 0.010989 | 0.148236 | 1 | 2.571429 | 0 |
| 2635 | 8 | 0.010989 | 0.148236 | 1 | 0.142857 | 16.42857 |
| 2690 | 10.5 | 0.010989 | 0.148236 | 1 | 0 | 1.285714 |
| 3626 | 6.5 | 0.010989 | 0.148236 | 1 | 3.428571 | 1 |
| 3688 | 10.5 | 0.010989 | 0.148236 | 1 | 0 | 1.285714 |
| 4053 | 10.5 | 0.010989 | 0.148236 | 1 | 1.428571 | 0 |
| 5115 | 10.5 | 0.010989 | 0.148236 | 1 | 1 | 0 |
| 5195 | 10.5 | 0.010989 | 0.148236 | 1 | 0.857143 | 0 |
| 5705 | 6.5 | 0.010989 | 0.148236 | 1 | 1.428571 | 10.14286 |
| 7995 | 6.5 | 0.010989 | 0.148236 | 1 | 5.714286 | 1.285714 |
| 229 | 6.5 | 0.011988 | 0.152692 | 1 | 0.428571 | 10.14286 |
| 450 | 8 | 0.011988 | 0.152692 | 1 | 13.85714 | 0.571429 |
| 545 | 5 | 0.011988 | 0.152692 | 1 | 52.28571 | 0.571429 |
| 969 | 10.5 | 0.011988 | 0.152692 | 1 | 0 | 1.142857 |
| 970 | 6.5 | 0.011988 | 0.152692 | 1 | 6.142857 | 1.714286 |
| 1129 | 6 | 0.011988 | 0.152692 | 1 | 58.57143 | 5.428571 |
| 1923 | 5.5 | 0.011988 | 0.152692 | 1 | 8.857143 | 1.142857 |
| 2272 | 10.5 | 0.011988 | 0.152692 | 1 | 0 | 0.714286 |
| 2421 | 10.5 | 0.011988 | 0.152692 | 1 | 0 | 1.428571 |
| 2515 | 10.5 | 0.011988 | 0.152692 | 1 | 0 | 1.571429 |
| 2837 | 7.5 | 0.011988 | 0.152692 | 1 | 0.285714 | 1.714286 |
| 3298 | 8.5 | 0.011988 | 0.152692 | 1 | 0.142857 | 8.857143 |
| 3342 | 10.5 | 0.011988 | 0.152692 | 1 | 6.857143 | 0 |
| 3698 | 7 | 0.011988 | 0.152692 | 1 | 34 | 65.14286 |
| 3699 | 10.5 | 0.011988 | 0.152692 | 1 | 0 | 0.857143 |
| 3804 | 10.5 | 0.011988 | 0.152692 | 1 | 0 | 0.857143 |
| 4817 | 10.5 | 0.011988 | 0.152692 | 1 | 1.142857 | 0 |
| 4966 | 7 | 0.011988 | 0.152692 | 1 | 2.428571 | 0.428571 |
| 4992 | 10.5 | 0.011988 | 0.152692 | 1 | 1.142857 | 0 |
| 5063 | 7 | 0.011988 | 0.152692 | 1 | 26.42857 | 2.142857 |
| 5394 | 10.5 | 0.011988 | 0.152692 | 1 | 0.714286 | 0 |
| 5549 | 6.5 | 0.011988 | 0.152692 | 1 | 0.857143 | 15.71429 |
| 6166 | 10.5 | 0.011988 | 0.152692 | 1 | 0 | 2.857143 |
| 6426 | 10.5 | 0.011988 | 0.152692 | 1 | 0 | 0.714286 |
| 6590 | 10.5 | 0.011988 | 0.152692 | 1 | 0 | 1.714286 |
| 7084 | 10.5 | 0.011988 | 0.152692 | 1 | 0 | 4.142857 |
| 7085 | 10.5 | 0.011988 | 0.152692 | 1 | 0 | 2.857143 |
| 7270 | 10.5 | 0.011988 | 0.152692 | 1 | 0 | 2.571429 |
| 43 | 6.5 | 0.012987 | 0.155504 | 1 | 7.857143 | 0.285714 |
| 443 | 7.5 | 0.012987 | 0.155504 | 1 | 9.428571 | 0.285714 |
| 575 | 6 | 0.012987 | 0.155504 | 1 | 18.28571 | 82.42857 |
| 601 | 6 | 0.012987 | 0.155504 | 1 | 20.71429 | 7.142857 |
| 796 | 14 | 0.012987 | 0.155504 | 1 | 0.428571 | 0 |
| 915 | 8.5 | 0.012987 | 0.155504 | 1 | 0.142857 | 1.571429 |
| 946 | 10.5 | 0.012987 | 0.155504 | 1 | 0 | 0.857143 |
| 1066 | 9 | 0.012987 | 0.155504 | 1 | 3.142857 | 0.142857 |
| 1158 | 9 | 0.012987 | 0.155504 | 1 | 0.285714 | 5.428571 |
| 1399 | 5.5 | 0.012987 | 0.155504 | 1 | 36.42857 | 7.428571 |
| 1424 | 7 | 0.012987 | 0.155504 | 1 | 1.571429 | 12.85714 |
| 1571 | 10.5 | 0.012987 | 0.155504 | 1 | 0 | 1.142857 |
| 1972 | 5.5 | 0.012987 | 0.155504 | 1 | 4.857143 | 41.85714 |
| 2074 | 7 | 0.012987 | 0.155504 | 1 | 0.857143 | 12.85714 |
| 2311 | 6 | 0.012987 | 0.155504 | 1 | 5.428571 | 27.57143 |
| 2383 | 10.5 | 0.012987 | 0.155504 | 1 | 2.285714 | 0 |
| 2531 | 10.5 | 0.012987 | 0.155504 | 1 | 0 | 3.428571 |
| 2667 | 6 | 0.012987 | 0.155504 | 1 | 15.28571 | 204.2857 |
| 2770 | 6.5 | 0.012987 | 0.155504 | 1 | 5.142857 | 0.428571 |
| 3032 | 10.5 | 0.012987 | 0.155504 | 1 | 0 | 1 |
| 3411 | 10.5 | 0.012987 | 0.155504 | 1 | 0 | 2 |
| 3810 | 5.5 | 0.012987 | 0.155504 | 1 | 0.285714 | 5.571429 |
| 3819 | 8.5 | 0.012987 | 0.155504 | 1 | 2 | 0.142857 |
| 3996 | 10.5 | 0.012987 | 0.155504 | 1 | 2.285714 | 0 |
| 4906 | 10.5 | 0.012987 | 0.155504 | 1 | 1.571429 | 0 |
| 4928 | 10.5 | 0.012987 | 0.155504 | 1 | 1.428571 | 0 |
| 5014 | 8 | 0.012987 | 0.155504 | 1 | 0.142857 | 4.285714 |
| 5473 | 6 | 0.012987 | 0.155504 | 1 | 0.857143 | 4.857143 |
| 6448 | 10.5 | 0.012987 | 0.155504 | 1 | 0 | 0.857143 |
| 6694 | 6.5 | 0.012987 | 0.155504 | 1 | 0.428571 | 6.428571 |
| 7785 | 7.5 | 0.012987 | 0.155504 | 1 | 0.285714 | 3.142857 |
| 8520 | 10.5 | 0.012987 | 0.155504 | 1 | 1.857143 | 0 |
| 56 | 6.5 | 0.013986 | 0.158558 | 1 | 18.42857 | 3.142857 |
| 570 | 6.5 | 0.013986 | 0.158558 | 1 | 0.428571 | 5.714286 |
| 701 | 9 | 0.013986 | 0.158558 | 1 | 0.142857 | 8 |
| 931 | 6.5 | 0.013986 | 0.158558 | 1 | 15 | 1.285714 |
| 1921 | 10.5 | 0.013986 | 0.158558 | 1 | 1.857143 | 0 |
| 2132 | 10.5 | 0.013986 | 0.158558 | 1 | 2.285714 | 0 |
| 2250 | 6 | 0.013986 | 0.158558 | 1 | 18.71429 | 1.857143 |
| 2278 | 5.5 | 0.013986 | 0.158558 | 1 | 4.857143 | 20 |
| 2410 | 5 | 0.013986 | 0.158558 | 1 | 7.285714 | 76.42857 |
| 2488 | 9 | 0.013986 | 0.158558 | 1 | 2.857143 | 0.285714 |
| 2519 | 7.5 | 0.013986 | 0.158558 | 1 | 1.857143 | 0.285714 |
| 3008 | 6 | 0.013986 | 0.158558 | 1 | 12.42857 | 1.857143 |
| 3412 | 8 | 0.013986 | 0.158558 | 1 | 0.142857 | 12 |
| 3511 | 10.5 | 0.013986 | 0.158558 | 1 | 0 | 1.142857 |
| 3627 | 10.5 | 0.013986 | 0.158558 | 1 | 0 | 0.857143 |
| 3675 | 10.5 | 0.013986 | 0.158558 | 1 | 0 | 0.714286 |
| 3809 | 7.5 | 0.013986 | 0.158558 | 1 | 2 | 0.285714 |
| 4004 | 9 | 0.013986 | 0.158558 | 1 | 0.142857 | 1.571429 |
| 4087 | 8 | 0.013986 | 0.158558 | 1 | 2.142857 | 0.142857 |
| 4247 | 14 | 0.013986 | 0.158558 | 1 | 0 | 0.428571 |
| 4496 | 9 | 0.013986 | 0.158558 | 1 | 1.285714 | 0.285714 |
| 4626 | 10.5 | 0.013986 | 0.158558 | 1 | 1.428571 | 0 |
| 5017 | 10.5 | 0.013986 | 0.158558 | 1 | 0.857143 | 0 |
| 6035 | 10.5 | 0.013986 | 0.158558 | 1 | 0 | 1.142857 |
| 6241 | 10.5 | 0.013986 | 0.158558 | 1 | 0 | 2.428571 |
| 6278 | 8 | 0.013986 | 0.158558 | 1 | 0.142857 | 5 |
| 6344 | 10.5 | 0.013986 | 0.158558 | 1 | 0 | 0.714286 |
| 6610 | 10.5 | 0.013986 | 0.158558 | 1 | 0 | 0.714286 |
| 6966 | 10.5 | 0.013986 | 0.158558 | 1 | 0 | 3.142857 |
| 8601 | 9.5 | 0.013986 | 0.158558 | 1 | 1.285714 | 0.142857 |
| 123 | 7.5 | 0.014985 | 0.159425 | 1 | 1.428571 | 16.42857 |
| 185 | 7 | 0.014985 | 0.159425 | 1 | 13 | 1.714286 |
| 669 | 7.5 | 0.014985 | 0.159425 | 1 | 6 | 1.142857 |
| 722 | 10.5 | 0.014985 | 0.159425 | 1 | 0 | 7 |
| 847 | 10.5 | 0.014985 | 0.159425 | 1 | 0 | 2 |
| 910 | 7.5 | 0.014985 | 0.159425 | 1 | 10.42857 | 27.71429 |
| 1607 | 8.5 | 0.014985 | 0.159425 | 1 | 1.142857 | 0.285714 |
| 2083 | 10.5 | 0.014985 | 0.159425 | 1 | 0 | 0.857143 |
| 2160 | 7.5 | 0.014985 | 0.159425 | 1 | 0.571429 | 4.142857 |
| 2312 | 9 | 0.014985 | 0.159425 | 1 | 4.428571 | 0.285714 |
| 2339 | 7 | 0.014985 | 0.159425 | 1 | 2.714286 | 0.428571 |
| 2341 | 6 | 0.014985 | 0.159425 | 1 | 8 | 1.857143 |
| 3015 | 9 | 0.014985 | 0.159425 | 1 | 0.285714 | 5.714286 |
| 3035 | 9 | 0.014985 | 0.159425 | 1 | 0.142857 | 2.428571 |
| 3074 | 8 | 0.014985 | 0.159425 | 1 | 1.571429 | 0.285714 |
| 3138 | 10.5 | 0.014985 | 0.159425 | 1 | 0 | 1 |
| 3474 | 10.5 | 0.014985 | 0.159425 | 1 | 0 | 1.142857 |
| 3483 | 6 | 0.014985 | 0.159425 | 1 | 0.571429 | 4 |
| 3634 | 10.5 | 0.014985 | 0.159425 | 1 | 1.142857 | 0 |
| 3652 | 10.5 | 0.014985 | 0.159425 | 1 | 0 | 1.142857 |
| 3856 | 10.5 | 0.014985 | 0.159425 | 1 | 7 | 0 |
| 3915 | 8 | 0.014985 | 0.159425 | 1 | 4.142857 | 0.571429 |
| 4195 | 10.5 | 0.014985 | 0.159425 | 1 | 0 | 1.142857 |
| 4236 | 10.5 | 0.014985 | 0.159425 | 1 | 0.857143 | 0 |
| 4412 | 10.5 | 0.014985 | 0.159425 | 1 | 0 | 0.857143 |
| 4434 | 7.5 | 0.014985 | 0.159425 | 1 | 6.285714 | 0.857143 |
| 4897 | 10.5 | 0.014985 | 0.159425 | 1 | 1.857143 | 0 |
| 4962 | 10.5 | 0.014985 | 0.159425 | 1 | 1.142857 | 0 |
| 4995 | 10.5 | 0.014985 | 0.159425 | 1 | 0 | 1.142857 |
| 5518 | 10.5 | 0.014985 | 0.159425 | 1 | 0 | 11.42857 |
| 5948 | 10.5 | 0.014985 | 0.159425 | 1 | 0.714286 | 0 |
| 6060 | 10.5 | 0.014985 | 0.159425 | 1 | 0 | 0.714286 |
| 6118 | 7.5 | 0.014985 | 0.159425 | 1 | 0.857143 | 10 |
| 6183 | 5.5 | 0.014985 | 0.159425 | 1 | 0.285714 | 4.857143 |
| 6322 | 10.5 | 0.014985 | 0.159425 | 1 | 0 | 0.714286 |
| 7380 | 7.5 | 0.014985 | 0.159425 | 1 | 3.285714 | 0.428571 |
| 8007 | 9 | 0.014985 | 0.159425 | 1 | 2.857143 | 0.142857 |
| 62 | 7 | 0.015984 | 0.163262 | 1 | 126.1429 | 27.71429 |
| 246 | 7 | 0.015984 | 0.163262 | 1 | 8.285714 | 142.1429 |
| 254 | 7 | 0.015984 | 0.163262 | 1 | 21.71429 | 54.28571 |
| 313 | 9 | 0.015984 | 0.163262 | 1 | 9.285714 | 0.142857 |
| 431 | 8.5 | 0.015984 | 0.163262 | 1 | 7.571429 | 0.142857 |
| 638 | 7.5 | 0.015984 | 0.163262 | 1 | 5.285714 | 0.571429 |
| 993 | 8.5 | 0.015984 | 0.163262 | 1 | 2.714286 | 0.285714 |
| 1184 | 8 | 0.015984 | 0.163262 | 1 | 0.142857 | 5.142857 |
| 1260 | 10.5 | 0.015984 | 0.163262 | 1 | 0 | 1.714286 |
| 1985 | 7.5 | 0.015984 | 0.163262 | 1 | 1.142857 | 22.14286 |
| 2123 | 10.5 | 0.015984 | 0.163262 | 1 | 3 | 0 |
| 2454 | 10.5 | 0.015984 | 0.163262 | 1 | 0 | 1 |
| 2637 | 8.5 | 0.015984 | 0.163262 | 1 | 0.142857 | 4.714286 |
| 2647 | 7 | 0.015984 | 0.163262 | 1 | 0.571429 | 9.571429 |
| 2877 | 7 | 0.015984 | 0.163262 | 1 | 0.428571 | 6.571429 |
| 3659 | 9 | 0.015984 | 0.163262 | 1 | 0.142857 | 1.857143 |
| 4024 | 10.5 | 0.015984 | 0.163262 | 1 | 1.142857 | 0 |
| 5339 | 10.5 | 0.015984 | 0.163262 | 1 | 0.714286 | 0 |
| 5700 | 10.5 | 0.015984 | 0.163262 | 1 | 0 | 1.142857 |
| 6415 | 9.5 | 0.015984 | 0.163262 | 1 | 0.142857 | 1.285714 |
| 6774 | 7 | 0.015984 | 0.163262 | 1 | 1.285714 | 4.142857 |
| 6798 | 10.5 | 0.015984 | 0.163262 | 1 | 0 | 0.857143 |
| 6995 | 9 | 0.015984 | 0.163262 | 1 | 0.142857 | 3.285714 |
| 7299 | 10.5 | 0.015984 | 0.163262 | 1 | 0 | 0.857143 |
| 7566 | 10.5 | 0.015984 | 0.163262 | 1 | 0 | 0.714286 |
| 35 | 6 | 0.016983 | 0.166039 | 1 | 1506.143 | 2689.143 |
| 266 | 7 | 0.016983 | 0.166039 | 1 | 1114.286 | 276.2857 |
| 685 | 10.5 | 0.016983 | 0.166039 | 1 | 3.428571 | 0 |
| 706 | 10.5 | 0.016983 | 0.166039 | 1 | 2.857143 | 0 |
| 955 | 6 | 0.016983 | 0.166039 | 1 | 9.285714 | 1.285714 |
| 1423 | 9 | 0.016983 | 0.166039 | 1 | 1.714286 | 0.142857 |
| 1621 | 10.5 | 0.016983 | 0.166039 | 1 | 0.714286 | 0 |
| 2315 | 8.5 | 0.016983 | 0.166039 | 1 | 2 | 0.285714 |
| 2370 | 14 | 0.016983 | 0.166039 | 1 | 0 | 0.428571 |
| 2439 | 8 | 0.016983 | 0.166039 | 1 | 0.285714 | 1.857143 |
| 2955 | 7.5 | 0.016983 | 0.166039 | 1 | 7.714286 | 0.571429 |
| 3702 | 14 | 0.016983 | 0.166039 | 1 | 0.428571 | 0 |
| 4020 | 8.5 | 0.016983 | 0.166039 | 1 | 0.285714 | 2.428571 |
| 4062 | 8.5 | 0.016983 | 0.166039 | 1 | 1.857143 | 0.428571 |
| 4232 | 8 | 0.016983 | 0.166039 | 1 | 4.428571 | 0.142857 |
| 4592 | 14 | 0.016983 | 0.166039 | 1 | 0.428571 | 0 |
| 4860 | 8 | 0.016983 | 0.166039 | 1 | 1.714286 | 5.142857 |
| 5047 | 14 | 0.016983 | 0.166039 | 1 | 0.428571 | 0 |
| 5435 | 14 | 0.016983 | 0.166039 | 1 | 0.428571 | 0 |
| 5632 | 10.5 | 0.016983 | 0.166039 | 1 | 0 | 1.428571 |
| 6057 | 10.5 | 0.016983 | 0.166039 | 1 | 0 | 1.571429 |
| 6227 | 10.5 | 0.016983 | 0.166039 | 1 | 0 | 1.142857 |
| 6483 | 10.5 | 0.016983 | 0.166039 | 1 | 0 | 1 |
| 6941 | 14 | 0.016983 | 0.166039 | 1 | 0 | 0.428571 |
| 6986 | 10.5 | 0.016983 | 0.166039 | 1 | 0 | 1 |
| 7457 | 10.5 | 0.016983 | 0.166039 | 1 | 0 | 1.428571 |
| 7560 | 10.5 | 0.016983 | 0.166039 | 1 | 0 | 1.571429 |
| 8664 | 10.5 | 0.016983 | 0.166039 | 1 | 2.142857 | 0 |
| 91 | 9 | 0.017982 | 0.168836 | 1 | 3 | 0.142857 |
| 409 | 6 | 0.017982 | 0.168836 | 1 | 12.85714 | 101.5714 |
| 542 | 6 | 0.017982 | 0.168836 | 1 | 9.857143 | 30.14286 |
| 544 | 6.5 | 0.017982 | 0.168836 | 1 | 1.285714 | 13.57143 |
| 653 | 10.5 | 0.017982 | 0.168836 | 1 | 2.142857 | 0 |
| 1146 | 7.5 | 0.017982 | 0.168836 | 1 | 1.142857 | 4.285714 |
| 1303 | 8.5 | 0.017982 | 0.168836 | 1 | 0.714286 | 2.428571 |
| 1312 | 9 | 0.017982 | 0.168836 | 1 | 4.571429 | 0.142857 |
| 1579 | 7 | 0.017982 | 0.168836 | 1 | 1.571429 | 20 |
| 1589 | 7 | 0.017982 | 0.168836 | 1 | 50 | 11.57143 |
| 2286 | 7 | 0.017982 | 0.168836 | 1 | 2.428571 | 15.42857 |
| 2970 | 9 | 0.017982 | 0.168836 | 1 | 56.42857 | 2.142857 |
| 3300 | 6.5 | 0.017982 | 0.168836 | 1 | 4.285714 | 36.42857 |
| 3500 | 14 | 0.017982 | 0.168836 | 1 | 0 | 0.428571 |
| 3541 | 8.5 | 0.017982 | 0.168836 | 1 | 0.142857 | 2.142857 |
| 3644 | 7.5 | 0.017982 | 0.168836 | 1 | 2.428571 | 6 |
| 3654 | 9 | 0.017982 | 0.168836 | 1 | 0.142857 | 1.571429 |
| 3886 | 8 | 0.017982 | 0.168836 | 1 | 0.428571 | 6.571429 |
| 4448 | 7.5 | 0.017982 | 0.168836 | 1 | 17.57143 | 1.428571 |
| 5370 | 8.5 | 0.017982 | 0.168836 | 1 | 0.142857 | 2.428571 |
| 5723 | 7 | 0.017982 | 0.168836 | 1 | 2.857143 | 10 |
| 5914 | 14 | 0.017982 | 0.168836 | 1 | 0.428571 | 0 |
| 6224 | 10.5 | 0.017982 | 0.168836 | 1 | 0 | 1.142857 |
| 6403 | 10.5 | 0.017982 | 0.168836 | 1 | 0 | 0.714286 |
| 6862 | 14 | 0.017982 | 0.168836 | 1 | 0 | 0.428571 |
| 6932 | 10.5 | 0.017982 | 0.168836 | 1 | 0 | 2.714286 |
| 7895 | 14 | 0.017982 | 0.168836 | 1 | 0 | 0.428571 |
| 117 | 7 | 0.018981 | 0.171419 | 1 | 710.4286 | 166.8571 |
| 639 | 8.5 | 0.018981 | 0.171419 | 1 | 0.285714 | 1.571429 |
| 756 | 6 | 0.018981 | 0.171419 | 1 | 21.14286 | 6.285714 |
| 1067 | 7.5 | 0.018981 | 0.171419 | 1 | 0.285714 | 6.428571 |
| 1103 | 9 | 0.018981 | 0.171419 | 1 | 0.285714 | 7.285714 |
| 1231 | 14 | 0.018981 | 0.171419 | 1 | 0.428571 | 0 |
| 1936 | 7.5 | 0.018981 | 0.171419 | 1 | 3.142857 | 0.428571 |
| 2007 | 7.5 | 0.018981 | 0.171419 | 1 | 3.428571 | 0.571429 |
| 2174 | 14 | 0.018981 | 0.171419 | 1 | 0.428571 | 0 |
| 2384 | 8 | 0.018981 | 0.171419 | 1 | 1.571429 | 8.428571 |
| 2436 | 8.5 | 0.018981 | 0.171419 | 1 | 3.714286 | 1 |
| 2764 | 7 | 0.018981 | 0.171419 | 1 | 0.571429 | 9.571429 |
| 2968 | 7.5 | 0.018981 | 0.171419 | 1 | 0.714286 | 3.714286 |
| 3470 | 14 | 0.018981 | 0.171419 | 1 | 0 | 0.428571 |
| 3484 | 14 | 0.018981 | 0.171419 | 1 | 0 | 0.428571 |
| 4025 | 9.5 | 0.018981 | 0.171419 | 1 | 1.571429 | 0.142857 |
| 4629 | 14 | 0.018981 | 0.171419 | 1 | 0.428571 | 0 |
| 4718 | 7 | 0.018981 | 0.171419 | 1 | 1.714286 | 7 |
| 5071 | 8.5 | 0.018981 | 0.171419 | 1 | 0.142857 | 3 |
| 5479 | 7.5 | 0.018981 | 0.171419 | 1 | 0.571429 | 2 |
| 5637 | 10.5 | 0.018981 | 0.171419 | 1 | 0 | 0.857143 |
| 6062 | 10.5 | 0.018981 | 0.171419 | 1 | 0 | 1.142857 |
| 6325 | 10.5 | 0.018981 | 0.171419 | 1 | 0 | 2.428571 |
| 6512 | 10.5 | 0.018981 | 0.171419 | 1 | 0 | 4.571429 |
| 6738 | 10.5 | 0.018981 | 0.171419 | 1 | 0 | 1 |
| 6828 | 10 | 0.018981 | 0.171419 | 1 | 0.142857 | 1 |
| 7178 | 10.5 | 0.018981 | 0.171419 | 1 | 0 | 1 |
| 205 | 8 | 0.01998 | 0.175725 | 1 | 10.28571 | 1.285714 |
| 220 | 14 | 0.01998 | 0.175725 | 1 | 0.428571 | 0 |
| 251 | 14 | 0.01998 | 0.175725 | 1 | 0.428571 | 0 |
| 368 | 9.5 | 0.01998 | 0.175725 | 1 | 0.285714 | 5.571429 |
| 487 | 7 | 0.01998 | 0.175725 | 1 | 21.28571 | 68.57143 |
| 547 | 10.5 | 0.01998 | 0.175725 | 1 | 0 | 1.142857 |
| 734 | 14 | 0.01998 | 0.175725 | 1 | 0 | 0.428571 |
| 811 | 7.5 | 0.01998 | 0.175725 | 1 | 1.428571 | 20.85714 |
| 1942 | 14 | 0.01998 | 0.175725 | 1 | 0.428571 | 0 |
| 2016 | 7 | 0.01998 | 0.175725 | 1 | 21.42857 | 14.42857 |
| 2221 | 8 | 0.01998 | 0.175725 | 1 | 7 | 0.571429 |
| 2423 | 6.5 | 0.01998 | 0.175725 | 1 | 1.428571 | 5.571429 |
| 2650 | 14 | 0.01998 | 0.175725 | 1 | 0.428571 | 0 |
| 2759 | 14 | 0.01998 | 0.175725 | 1 | 0 | 0.428571 |
| 3203 | 14 | 0.01998 | 0.175725 | 1 | 0.428571 | 0 |
| 3946 | 7.5 | 0.01998 | 0.175725 | 1 | 2.142857 | 6.714286 |
| 4319 | 10.5 | 0.01998 | 0.175725 | 1 | 1.142857 | 0 |
| 4637 | 7 | 0.01998 | 0.175725 | 1 | 4.428571 | 1 |
| 6781 | 10.5 | 0.01998 | 0.175725 | 1 | 0 | 2.571429 |
| 81 | 7.5 | 0.020979 | 0.178853 | 1 | 1.714286 | 8.428571 |
| 458 | 7 | 0.020979 | 0.178853 | 1 | 34.42857 | 159.2857 |
| 808 | 7.5 | 0.020979 | 0.178853 | 1 | 11.14286 | 1.571429 |
| 1054 | 6.5 | 0.020979 | 0.178853 | 1 | 31.42857 | 1.428571 |
| 1121 | 7 | 0.020979 | 0.178853 | 1 | 10.42857 | 31.85714 |
| 2092 | 14 | 0.020979 | 0.178853 | 1 | 0 | 0.428571 |
| 2212 | 8 | 0.020979 | 0.178853 | 1 | 3.571429 | 21.85714 |
| 2386 | 9 | 0.020979 | 0.178853 | 1 | 0.428571 | 3.428571 |
| 2880 | 10.5 | 0.020979 | 0.178853 | 1 | 5.428571 | 0 |
| 3646 | 10 | 0.020979 | 0.178853 | 1 | 0.142857 | 1.142857 |
| 3913 | 8 | 0.020979 | 0.178853 | 1 | 3 | 11.42857 |
| 4106 | 6.5 | 0.020979 | 0.178853 | 1 | 1.714286 | 7.285714 |
| 4164 | 9 | 0.020979 | 0.178853 | 1 | 1.714286 | 0.142857 |
| 4473 | 14 | 0.020979 | 0.178853 | 1 | 0.428571 | 0 |
| 4550 | 14 | 0.020979 | 0.178853 | 1 | 0 | 0.428571 |
| 4885 | 7.5 | 0.020979 | 0.178853 | 1 | 4.857143 | 0.571429 |
| 5296 | 9.5 | 0.020979 | 0.178853 | 1 | 0.285714 | 3 |
| 5770 | 8 | 0.020979 | 0.178853 | 1 | 6.571429 | 1.714286 |
| 6237 | 9.5 | 0.020979 | 0.178853 | 1 | 0.142857 | 3.714286 |
| 6282 | 14 | 0.020979 | 0.178853 | 1 | 0 | 0.428571 |
| 6627 | 9 | 0.020979 | 0.178853 | 1 | 0.142857 | 2.285714 |
| 9051 | 14 | 0.020979 | 0.178853 | 1 | 0.428571 | 0 |
| 9055 | 14 | 0.020979 | 0.178853 | 1 | 0 | 0.428571 |
| 36 | 7.5 | 0.021978 | 0.183456 | 1 | 17.14286 | 3.857143 |
| 110 | 8 | 0.021978 | 0.183456 | 1 | 15.28571 | 0.428571 |
| 737 | 9 | 0.021978 | 0.183456 | 1 | 0.142857 | 3.714286 |
| 790 | 6.5 | 0.021978 | 0.183456 | 1 | 28.28571 | 1.142857 |
| 935 | 8.5 | 0.021978 | 0.183456 | 1 | 0.142857 | 5 |
| 984 | 7.5 | 0.021978 | 0.183456 | 1 | 13.42857 | 2.857143 |
| 1088 | 9 | 0.021978 | 0.183456 | 1 | 0.142857 | 1.428571 |
| 1987 | 8 | 0.021978 | 0.183456 | 1 | 5 | 0.571429 |
| 2108 | 9 | 0.021978 | 0.183456 | 1 | 0.714286 | 4 |
| 2660 | 8 | 0.021978 | 0.183456 | 1 | 2.857143 | 1.571429 |
| 3256 | 10 | 0.021978 | 0.183456 | 1 | 0.857143 | 0.142857 |
| 4372 | 8 | 0.021978 | 0.183456 | 1 | 13 | 4.142857 |
| 4823 | 8.5 | 0.021978 | 0.183456 | 1 | 2 | 7.285714 |
| 6720 | 14 | 0.021978 | 0.183456 | 1 | 0 | 0.571429 |
| 8504 | 14 | 0.021978 | 0.183456 | 1 | 0 | 0.428571 |
| 8744 | 14 | 0.021978 | 0.183456 | 1 | 0.428571 | 0 |
| 332 | 8 | 0.022977 | 0.188112 | 1 | 10.71429 | 28.42857 |
| 878 | 10 | 0.022977 | 0.188112 | 1 | 0.285714 | 3.857143 |
| 1350 | 7.5 | 0.022977 | 0.188112 | 1 | 2.285714 | 25.71429 |
| 1603 | 8 | 0.022977 | 0.188112 | 1 | 24.42857 | 56 |
| 1612 | 10 | 0.022977 | 0.188112 | 1 | 0.857143 | 0.142857 |
| 1628 | 8.5 | 0.022977 | 0.188112 | 1 | 0.714286 | 24 |
| 3922 | 14 | 0.022977 | 0.188112 | 1 | 0 | 0.428571 |
| 4069 | 7 | 0.022977 | 0.188112 | 1 | 75.14286 | 11.28571 |
| 4276 | 10.5 | 0.022977 | 0.188112 | 1 | 1 | 0 |
| 4454 | 14 | 0.022977 | 0.188112 | 1 | 0 | 0.428571 |
| 5364 | 9.5 | 0.022977 | 0.188112 | 1 | 3.285714 | 0.857143 |
| 5711 | 14 | 0.022977 | 0.188112 | 1 | 0 | 0.428571 |
| 6417 | 14 | 0.022977 | 0.188112 | 1 | 0 | 0.714286 |
| 6648 | 14 | 0.022977 | 0.188112 | 1 | 0 | 0.428571 |
| 7328 | 9 | 0.022977 | 0.188112 | 1 | 3.428571 | 12.85714 |
| 511 | 14 | 0.023976 | 0.192834 | 1 | 0 | 0.428571 |
| 740 | 8 | 0.023976 | 0.192834 | 1 | 13 | 2.571429 |
| 1093 | 9.5 | 0.023976 | 0.192834 | 1 | 0.142857 | 1.714286 |
| 1657 | 14 | 0.023976 | 0.192834 | 1 | 0 | 0.428571 |
| 2022 | 10 | 0.023976 | 0.192834 | 1 | 0.142857 | 0.857143 |
| 2233 | 8 | 0.023976 | 0.192834 | 1 | 1.285714 | 5.428571 |
| 2335 | 6.5 | 0.023976 | 0.192834 | 1 | 1.142857 | 5.857143 |
| 2487 | 10 | 0.023976 | 0.192834 | 1 | 0.857143 | 0.142857 |
| 2863 | 7.5 | 0.023976 | 0.192834 | 1 | 1.142857 | 6.285714 |
| 3970 | 10 | 0.023976 | 0.192834 | 1 | 1 | 0.142857 |
| 5780 | 14 | 0.023976 | 0.192834 | 1 | 0 | 0.571429 |
| 6767 | 10.5 | 0.023976 | 0.192834 | 1 | 0 | 0.857143 |
| 7789 | 8 | 0.023976 | 0.192834 | 1 | 1.571429 | 9.142857 |
| 8203 | 9.5 | 0.023976 | 0.192834 | 1 | 0.142857 | 1.142857 |
| 287 | 14 | 0.024975 | 0.197149 | 1 | 0 | 0.428571 |
| 391 | 14 | 0.024975 | 0.197149 | 1 | 0.428571 | 0 |
| 621 | 10 | 0.024975 | 0.197149 | 1 | 1.428571 | 0.142857 |
| 998 | 9.5 | 0.024975 | 0.197149 | 1 | 0.142857 | 1.857143 |
| 1980 | 7.5 | 0.024975 | 0.197149 | 1 | 3.857143 | 0.714286 |
| 2508 | 8.5 | 0.024975 | 0.197149 | 1 | 7 | 1.142857 |
| 2950 | 7.5 | 0.024975 | 0.197149 | 1 | 3.142857 | 0.571429 |
| 3859 | 14 | 0.024975 | 0.197149 | 1 | 0.428571 | 0 |
| 4039 | 8.5 | 0.024975 | 0.197149 | 1 | 0.285714 | 2.142857 |
| 5431 | 10 | 0.024975 | 0.197149 | 1 | 0.857143 | 0.142857 |
| 5699 | 14 | 0.024975 | 0.197149 | 1 | 0 | 0.714286 |
| 6481 | 14 | 0.024975 | 0.197149 | 1 | 0.428571 | 0 |
| 7419 | 14 | 0.024975 | 0.197149 | 1 | 0 | 1.285714 |
| 8401 | 14 | 0.024975 | 0.197149 | 1 | 0 | 0.714286 |
| 8535 | 14 | 0.024975 | 0.197149 | 1 | 0.428571 | 0 |
| 257 | 8 | 0.025974 | 0.199613 | 1 | 64.28571 | 128.2857 |
| 560 | 14 | 0.025974 | 0.199613 | 1 | 0.714286 | 0 |
| 635 | 14 | 0.025974 | 0.199613 | 1 | 0 | 0.571429 |
| 1570 | 9 | 0.025974 | 0.199613 | 1 | 3.857143 | 0.142857 |
| 2503 | 8 | 0.025974 | 0.199613 | 1 | 3.857143 | 11.57143 |
| 2669 | 14 | 0.025974 | 0.199613 | 1 | 0.857143 | 0 |
| 2888 | 8.5 | 0.025974 | 0.199613 | 1 | 0.142857 | 2.285714 |
| 2956 | 14 | 0.025974 | 0.199613 | 1 | 2.285714 | 0 |
| 3070 | 8 | 0.025974 | 0.199613 | 1 | 1.571429 | 4.571429 |
| 3426 | 8.5 | 0.025974 | 0.199613 | 1 | 4.857143 | 73 |
| 3832 | 8 | 0.025974 | 0.199613 | 1 | 1.142857 | 5.571429 |
| 3876 | 14 | 0.025974 | 0.199613 | 1 | 0.571429 | 0 |
| 3975 | 9 | 0.025974 | 0.199613 | 1 | 4.285714 | 0.285714 |
| 4834 | 14 | 0.025974 | 0.199613 | 1 | 0.571429 | 0 |
| 5107 | 9 | 0.025974 | 0.199613 | 1 | 3.714286 | 0.285714 |
| 5248 | 7.5 | 0.025974 | 0.199613 | 1 | 4 | 30 |
| 5681 | 9.5 | 0.025974 | 0.199613 | 1 | 0.142857 | 2.142857 |
| 5736 | 14 | 0.025974 | 0.199613 | 1 | 0 | 0.428571 |
| 6429 | 14 | 0.025974 | 0.199613 | 1 | 0 | 0.428571 |
| 6618 | 14 | 0.025974 | 0.199613 | 1 | 0 | 0.428571 |
| 6707 | 9.5 | 0.025974 | 0.199613 | 1 | 0.142857 | 1 |
| 7370 | 14 | 0.025974 | 0.199613 | 1 | 0 | 0.428571 |
| 518 | 8 | 0.026973 | 0.203379 | 1 | 59.85714 | 12.57143 |
| 546 | 14 | 0.026973 | 0.203379 | 1 | 0.714286 | 0 |
| 1092 | 14 | 0.026973 | 0.203379 | 1 | 1 | 0 |
| 1392 | 10 | 0.026973 | 0.203379 | 1 | 0.428571 | 3.428571 |
| 2295 | 9 | 0.026973 | 0.203379 | 1 | 0.857143 | 19 |
| 3615 | 14 | 0.026973 | 0.203379 | 1 | 0 | 0.714286 |
| 4475 | 14 | 0.026973 | 0.203379 | 1 | 0.428571 | 0 |
| 4914 | 14 | 0.026973 | 0.203379 | 1 | 0.571429 | 0 |
| 5361 | 14 | 0.026973 | 0.203379 | 1 | 0.857143 | 0 |
| 6172 | 14 | 0.026973 | 0.203379 | 1 | 0 | 1.142857 |
| 6949 | 9 | 0.026973 | 0.203379 | 1 | 2.857143 | 5.714286 |
| 7158 | 14 | 0.026973 | 0.203379 | 1 | 0 | 0.571429 |
| 7365 | 14 | 0.026973 | 0.203379 | 1 | 0 | 0.571429 |
| 7715 | 14 | 0.026973 | 0.203379 | 1 | 1.142857 | 0 |
| 8032 | 14 | 0.026973 | 0.203379 | 1 | 0.714286 | 0 |
| 8487 | 14 | 0.026973 | 0.203379 | 1 | 0 | 0.428571 |
| 239 | 9 | 0.027972 | 0.205342 | 1 | 14.57143 | 3.571429 |
| 250 | 8 | 0.027972 | 0.205342 | 1 | 4.571429 | 11.28571 |
| 902 | 9 | 0.027972 | 0.205342 | 1 | 0.285714 | 6.428571 |
| 925 | 8.5 | 0.027972 | 0.205342 | 1 | 12.14286 | 3.142857 |
| 1426 | 8.5 | 0.027972 | 0.205342 | 1 | 8.571429 | 40.28571 |
| 1642 | 8.5 | 0.027972 | 0.205342 | 1 | 5.857143 | 0.571429 |
| 2235 | 10 | 0.027972 | 0.205342 | 1 | 4.142857 | 0.285714 |
| 2364 | 8.5 | 0.027972 | 0.205342 | 1 | 6.571429 | 0.857143 |
| 2624 | 7.5 | 0.027972 | 0.205342 | 1 | 4.285714 | 12.57143 |
| 2813 | 14 | 0.027972 | 0.205342 | 1 | 1.428571 | 0 |
| 3516 | 14 | 0.027972 | 0.205342 | 1 | 0 | 0.714286 |
| 3645 | 8 | 0.027972 | 0.205342 | 1 | 3.714286 | 10.42857 |
| 3893 | 8 | 0.027972 | 0.205342 | 1 | 1.142857 | 6.142857 |
| 4348 | 9.5 | 0.027972 | 0.205342 | 1 | 0.142857 | 1.142857 |
| 4501 | 14 | 0.027972 | 0.205342 | 1 | 0.714286 | 0 |
| 5981 | 14 | 0.027972 | 0.205342 | 1 | 0 | 1.142857 |
| 6015 | 14 | 0.027972 | 0.205342 | 1 | 0 | 1.714286 |
| 6079 | 14 | 0.027972 | 0.205342 | 1 | 0 | 0.571429 |
| 6175 | 14 | 0.027972 | 0.205342 | 1 | 0 | 0.857143 |
| 6716 | 14 | 0.027972 | 0.205342 | 1 | 0 | 1.142857 |
| 6905 | 14 | 0.027972 | 0.205342 | 1 | 0 | 2 |
| 7777 | 14 | 0.027972 | 0.205342 | 1 | 0 | 0.428571 |
| 8908 | 14 | 0.027972 | 0.205342 | 1 | 0 | 0.714286 |
| 350 | 8.5 | 0.028971 | 0.209548 | 1 | 1.571429 | 11.85714 |
| 462 | 8 | 0.028971 | 0.209548 | 1 | 52.14286 | 12.28571 |
| 1219 | 14 | 0.028971 | 0.209548 | 1 | 1.142857 | 0 |
| 3807 | 14 | 0.028971 | 0.209548 | 1 | 0 | 0.571429 |
| 4066 | 14 | 0.028971 | 0.209548 | 1 | 0.571429 | 0 |
| 4067 | 8.5 | 0.028971 | 0.209548 | 1 | 0.571429 | 3.571429 |
| 4306 | 14 | 0.028971 | 0.209548 | 1 | 1 | 0 |
| 4932 | 14 | 0.028971 | 0.209548 | 1 | 0.857143 | 0 |
| 6130 | 14 | 0.028971 | 0.209548 | 1 | 0 | 0.428571 |
| 6598 | 10 | 0.028971 | 0.209548 | 1 | 0.285714 | 2 |
| 6887 | 14 | 0.028971 | 0.209548 | 1 | 0 | 1.142857 |
| 7068 | 17.5 | 0.028971 | 0.209548 | 1 | 0 | 0.285714 |
| 8512 | 10 | 0.028971 | 0.209548 | 1 | 0.571429 | 8.571429 |
| 241 | 14 | 0.02997 | 0.211978 | 1 | 0.428571 | 0 |
| 315 | 8.5 | 0.02997 | 0.211978 | 1 | 1.428571 | 306 |
| 629 | 9 | 0.02997 | 0.211978 | 1 | 0.714286 | 3.857143 |
| 1060 | 9 | 0.02997 | 0.211978 | 1 | 2.857143 | 0.285714 |
| 1344 | 14 | 0.02997 | 0.211978 | 1 | 0 | 0.714286 |
| 1959 | 14 | 0.02997 | 0.211978 | 1 | 0 | 0.857143 |
| 2079 | 8 | 0.02997 | 0.211978 | 1 | 0.857143 | 6.714286 |
| 2401 | 10 | 0.02997 | 0.211978 | 1 | 3 | 1.714286 |
| 2554 | 10.5 | 0.02997 | 0.211978 | 1 | 0.285714 | 2.142857 |
| 2636 | 8 | 0.02997 | 0.211978 | 1 | 267.1429 | 109.4286 |
| 3989 | 14 | 0.02997 | 0.211978 | 1 | 0 | 1.285714 |
| 4545 | 14 | 0.02997 | 0.211978 | 1 | 0.428571 | 0 |
| 4606 | 14 | 0.02997 | 0.211978 | 1 | 0.857143 | 0 |
| 4881 | 14 | 0.02997 | 0.211978 | 1 | 1 | 0 |
| 4911 | 10 | 0.02997 | 0.211978 | 1 | 0.285714 | 2.428571 |
| 5133 | 14 | 0.02997 | 0.211978 | 1 | 0.857143 | 0 |
| 6612 | 14 | 0.02997 | 0.211978 | 1 | 0 | 0.571429 |
| 6778 | 8 | 0.02997 | 0.211978 | 1 | 6 | 1.571429 |
| 7375 | 14 | 0.02997 | 0.211978 | 1 | 0 | 0.571429 |
| 9039 | 8 | 0.02997 | 0.211978 | 1 | 3.428571 | 8.857143 |
| 558 | 14 | 0.030969 | 0.213037 | 1 | 0 | 1 |
| 768 | 8 | 0.030969 | 0.213037 | 1 | 5.428571 | 13.28571 |
| 1316 | 7.5 | 0.030969 | 0.213037 | 1 | 5.714286 | 14.71429 |
| 2023 | 10 | 0.030969 | 0.213037 | 1 | 2.857143 | 0.285714 |
| 2399 | 8 | 0.030969 | 0.213037 | 1 | 3 | 47.28571 |
| 2894 | 14 | 0.030969 | 0.213037 | 1 | 0 | 0.857143 |
| 3786 | 14 | 0.030969 | 0.213037 | 1 | 1.285714 | 0 |
| 4204 | 9 | 0.030969 | 0.213037 | 1 | 2.142857 | 0.714286 |
| 4308 | 10.5 | 0.030969 | 0.213037 | 1 | 0.285714 | 1.571429 |
| 4516 | 14 | 0.030969 | 0.213037 | 1 | 0 | 0.714286 |
| 4809 | 14 | 0.030969 | 0.213037 | 1 | 0.571429 | 0 |
| 5280 | 9.5 | 0.030969 | 0.213037 | 1 | 0.142857 | 1.428571 |
| 6180 | 8 | 0.030969 | 0.213037 | 1 | 1.428571 | 8.857143 |
| 6312 | 9.5 | 0.030969 | 0.213037 | 1 | 0.571429 | 2.428571 |
| 6384 | 14 | 0.030969 | 0.213037 | 1 | 0 | 0.857143 |
| 6535 | 14 | 0.030969 | 0.213037 | 1 | 0 | 0.571429 |
| 6573 | 14 | 0.030969 | 0.213037 | 1 | 0 | 2.285714 |
| 6602 | 14 | 0.030969 | 0.213037 | 1 | 0 | 1.857143 |
| 6662 | 14 | 0.030969 | 0.213037 | 1 | 0 | 1.428571 |
| 7117 | 14 | 0.030969 | 0.213037 | 1 | 0 | 1 |
| 7679 | 10 | 0.030969 | 0.213037 | 1 | 0.428571 | 4.714286 |
| 7836 | 14 | 0.030969 | 0.213037 | 1 | 0 | 2.571429 |
| 8009 | 10 | 0.030969 | 0.213037 | 1 | 0.285714 | 2.142857 |
| 1846 | 14 | 0.031968 | 0.213037 | 1 | 0 | 0.571429 |
| 2317 | 8.5 | 0.031968 | 0.213037 | 1 | 2 | 4.714286 |
| 2559 | 14 | 0.031968 | 0.213037 | 1 | 0 | 1.285714 |
| 2627 | 14 | 0.031968 | 0.213037 | 1 | 2 | 0 |
| 2844 | 14 | 0.031968 | 0.213037 | 1 | 1.571429 | 0 |
| 3165 | 14 | 0.031968 | 0.213037 | 1 | 1 | 0 |
| 3803 | 14 | 0.031968 | 0.213037 | 1 | 0 | 1.428571 |
| 4660 | 10 | 0.031968 | 0.213037 | 1 | 6.428571 | 0.428571 |
| 4713 | 10 | 0.031968 | 0.213037 | 1 | 0.285714 | 4.285714 |
| 6293 | 14 | 0.031968 | 0.213037 | 1 | 0 | 0.571429 |
| 6386 | 14 | 0.031968 | 0.213037 | 1 | 0 | 1.285714 |
| 6929 | 14 | 0.031968 | 0.213037 | 1 | 0 | 0.857143 |
| 7162 | 14 | 0.031968 | 0.213037 | 1 | 0 | 0.571429 |
| 26 | 8 | 0.032967 | 0.213037 | 1 | 272.2857 | 18.28571 |
| 273 | 14 | 0.032967 | 0.213037 | 1 | 1 | 0 |
| 735 | 14 | 0.032967 | 0.213037 | 1 | 0.857143 | 0 |
| 794 | 14 | 0.032967 | 0.213037 | 1 | 0.714286 | 0 |
| 2964 | 14 | 0.032967 | 0.213037 | 1 | 1.714286 | 0 |
| 3503 | 14 | 0.032967 | 0.213037 | 1 | 0 | 2.571429 |
| 3596 | 14 | 0.032967 | 0.213037 | 1 | 0 | 1.142857 |
| 3705 | 8.5 | 0.032967 | 0.213037 | 1 | 3.142857 | 9.285714 |
| 3956 | 8.5 | 0.032967 | 0.213037 | 1 | 1.857143 | 14 |
| 4226 | 14 | 0.032967 | 0.213037 | 1 | 4 | 0 |
| 4274 | 14 | 0.032967 | 0.213037 | 1 | 0.857143 | 0 |
| 5656 | 9.5 | 0.032967 | 0.213037 | 1 | 1 | 6.857143 |
| 6311 | 9.5 | 0.032967 | 0.213037 | 1 | 0.857143 | 3.142857 |
| 6645 | 10 | 0.032967 | 0.213037 | 1 | 0.285714 | 28 |
| 8040 | 14 | 0.032967 | 0.213037 | 1 | 0 | 0.571429 |
| 8657 | 9.5 | 0.032967 | 0.213037 | 1 | 1 | 4.285714 |
| 481 | 9 | 0.033966 | 0.213037 | 1 | 10.71429 | 1 |
| 1113 | 14 | 0.033966 | 0.213037 | 1 | 2 | 0 |
| 1196 | 14 | 0.033966 | 0.213037 | 1 | 0 | 0.571429 |
| 1245 | 14 | 0.033966 | 0.213037 | 1 | 1.285714 | 0 |
| 1285 | 14 | 0.033966 | 0.213037 | 1 | 0 | 2.714286 |
| 1378 | 14 | 0.033966 | 0.213037 | 1 | 0 | 1.285714 |
| 2161 | 14 | 0.033966 | 0.213037 | 1 | 0.714286 | 0 |
| 2446 | 14 | 0.033966 | 0.213037 | 1 | 0 | 2.285714 |
| 3366 | 14 | 0.033966 | 0.213037 | 1 | 0 | 1 |
| 3777 | 14 | 0.033966 | 0.213037 | 1 | 0 | 116.1429 |
| 3894 | 14 | 0.033966 | 0.213037 | 1 | 3 | 0 |
| 4530 | 14 | 0.033966 | 0.213037 | 1 | 0 | 0.714286 |
| 4564 | 14 | 0.033966 | 0.213037 | 1 | 0.571429 | 0 |
| 4577 | 14 | 0.033966 | 0.213037 | 1 | 0 | 3 |
| 5163 | 14 | 0.033966 | 0.213037 | 1 | 0.857143 | 0 |
| 5689 | 14 | 0.033966 | 0.213037 | 1 | 0.714286 | 0 |
| 5953 | 14 | 0.033966 | 0.213037 | 1 | 0 | 0.857143 |
| 5956 | 9.5 | 0.033966 | 0.213037 | 1 | 5.285714 | 0.714286 |
| 6375 | 14 | 0.033966 | 0.213037 | 1 | 0 | 0.571429 |
| 6428 | 14 | 0.033966 | 0.213037 | 1 | 0 | 1.571429 |
| 6490 | 14 | 0.033966 | 0.213037 | 1 | 0 | 0.571429 |
| 6672 | 9.5 | 0.033966 | 0.213037 | 1 | 1.714286 | 6.857143 |
| 6736 | 14 | 0.033966 | 0.213037 | 1 | 0 | 1.428571 |
| 6893 | 14 | 0.033966 | 0.213037 | 1 | 0 | 0.571429 |
| 7377 | 14 | 0.033966 | 0.213037 | 1 | 0 | 1.714286 |
| 7569 | 14 | 0.033966 | 0.213037 | 1 | 0.571429 | 0 |
| 8153 | 14 | 0.033966 | 0.213037 | 1 | 0.571429 | 0 |
| 8434 | 14 | 0.033966 | 0.213037 | 1 | 0 | 0.571429 |
| 228 | 9 | 0.034965 | 0.213037 | 1 | 24.85714 | 47.14286 |
| 503 | 7.5 | 0.034965 | 0.213037 | 1 | 9.142857 | 17.85714 |
| 1404 | 14 | 0.034965 | 0.213037 | 1 | 0.857143 | 0 |
| 1539 | 9 | 0.034965 | 0.213037 | 1 | 2.142857 | 5 |
| 2089 | 14 | 0.034965 | 0.213037 | 1 | 0 | 2.571429 |
| 2415 | 14 | 0.034965 | 0.213037 | 1 | 0 | 1.142857 |
| 2602 | 9 | 0.034965 | 0.213037 | 1 | 1.571429 | 5 |
| 2936 | 14 | 0.034965 | 0.213037 | 1 | 0 | 1.571429 |
| 3018 | 14 | 0.034965 | 0.213037 | 1 | 0.714286 | 0 |
| 3271 | 14 | 0.034965 | 0.213037 | 1 | 0.857143 | 0 |
| 3923 | 14 | 0.034965 | 0.213037 | 1 | 0 | 0.571429 |
| 5020 | 10.5 | 0.034965 | 0.213037 | 1 | 7 | 0.714286 |
| 5508 | 14 | 0.034965 | 0.213037 | 1 | 0.571429 | 0 |
| 6364 | 14 | 0.034965 | 0.213037 | 1 | 0 | 0.571429 |
| 6623 | 14 | 0.034965 | 0.213037 | 1 | 0 | 0.571429 |
| 6686 | 10 | 0.034965 | 0.213037 | 1 | 0.285714 | 3.142857 |
| 6697 | 14 | 0.034965 | 0.213037 | 1 | 0 | 0.714286 |
| 7129 | 14 | 0.034965 | 0.213037 | 1 | 0 | 1 |
| 7275 | 14 | 0.034965 | 0.213037 | 1 | 0 | 0.571429 |
| 7276 | 14 | 0.034965 | 0.213037 | 1 | 0 | 0.714286 |
| 7804 | 14 | 0.034965 | 0.213037 | 1 | 0 | 8.571429 |
| 7918 | 14 | 0.034965 | 0.213037 | 1 | 0 | 0.857143 |
| 700 | 14 | 0.035964 | 0.213037 | 1 | 0 | 0.857143 |
| 1083 | 9 | 0.035964 | 0.213037 | 1 | 0.714286 | 4.857143 |
| 1721 | 14 | 0.035964 | 0.213037 | 1 | 1 | 0 |
| 1916 | 8.5 | 0.035964 | 0.213037 | 1 | 1.428571 | 10.28571 |
| 2232 | 9 | 0.035964 | 0.213037 | 1 | 7.571429 | 7.857143 |
| 2234 | 9 | 0.035964 | 0.213037 | 1 | 7.428571 | 1.142857 |
| 2407 | 14 | 0.035964 | 0.213037 | 1 | 0 | 0.857143 |
| 2963 | 14 | 0.035964 | 0.213037 | 1 | 0 | 0.571429 |
| 3082 | 14 | 0.035964 | 0.213037 | 1 | 1.285714 | 0 |
| 3345 | 14 | 0.035964 | 0.213037 | 1 | 0.857143 | 0 |
| 4176 | 14 | 0.035964 | 0.213037 | 1 | 0.857143 | 0 |
| 4540 | 14 | 0.035964 | 0.213037 | 1 | 0 | 0.571429 |
| 4742 | 14 | 0.035964 | 0.213037 | 1 | 0.857143 | 0 |
| 5603 | 14 | 0.035964 | 0.213037 | 1 | 0 | 0.714286 |
| 6048 | 14 | 0.035964 | 0.213037 | 1 | 0 | 0.714286 |
| 6630 | 17.5 | 0.035964 | 0.213037 | 1 | 0 | 1.428571 |
| 6696 | 14 | 0.035964 | 0.213037 | 1 | 0 | 0.857143 |
| 7500 | 14 | 0.035964 | 0.213037 | 1 | 0 | 1.857143 |
| 8019 | 14 | 0.035964 | 0.213037 | 1 | 0 | 0.571429 |
| 179 | 9 | 0.036963 | 0.213037 | 1 | 2.857143 | 0.571429 |
| 351 | 14 | 0.036963 | 0.213037 | 1 | 3.285714 | 0 |
| 396 | 10 | 0.036963 | 0.213037 | 1 | 3.285714 | 0.428571 |
| 926 | 10 | 0.036963 | 0.213037 | 1 | 3.714286 | 0.285714 |
| 1327 | 14 | 0.036963 | 0.213037 | 1 | 1.142857 | 0 |
| 1788 | 14 | 0.036963 | 0.213037 | 1 | 0 | 1.142857 |
| 1994 | 9 | 0.036963 | 0.213037 | 1 | 8.857143 | 38.42857 |
| 2131 | 14 | 0.036963 | 0.213037 | 1 | 0.857143 | 0 |
| 2346 | 14 | 0.036963 | 0.213037 | 1 | 0 | 0.857143 |
| 3546 | 14 | 0.036963 | 0.213037 | 1 | 0 | 1.142857 |
| 3595 | 14 | 0.036963 | 0.213037 | 1 | 0.857143 | 0 |
| 4099 | 9 | 0.036963 | 0.213037 | 1 | 0.714286 | 5.857143 |
| 4433 | 14 | 0.036963 | 0.213037 | 1 | 1 | 0 |
| 5043 | 14 | 0.036963 | 0.213037 | 1 | 1.428571 | 0 |
| 6228 | 14 | 0.036963 | 0.213037 | 1 | 0 | 3.428571 |
| 6575 | 14 | 0.036963 | 0.213037 | 1 | 0 | 0.571429 |
| 6643 | 14 | 0.036963 | 0.213037 | 1 | 0 | 1.571429 |
| 6976 | 14 | 0.036963 | 0.213037 | 1 | 0 | 0.714286 |
| 9148 | 14 | 0.036963 | 0.213037 | 1 | 0 | 0.571429 |
| 604 | 8.5 | 0.037962 | 0.213037 | 1 | 8.714286 | 1.857143 |
| 2472 | 14 | 0.037962 | 0.213037 | 1 | 0 | 1.714286 |
| 3220 | 14 | 0.037962 | 0.213037 | 1 | 0 | 4.142857 |
| 3524 | 17.5 | 0.037962 | 0.213037 | 1 | 0 | 0.285714 |
| 4346 | 14 | 0.037962 | 0.213037 | 1 | 0 | 1.714286 |
| 4367 | 9.5 | 0.037962 | 0.213037 | 1 | 1.571429 | 4.571429 |
| 4415 | 14 | 0.037962 | 0.213037 | 1 | 0.857143 | 0 |
| 4527 | 14 | 0.037962 | 0.213037 | 1 | 0.571429 | 0 |
| 4682 | 14 | 0.037962 | 0.213037 | 1 | 0.714286 | 0 |
| 4748 | 14 | 0.037962 | 0.213037 | 1 | 1 | 0 |
| 5085 | 14 | 0.037962 | 0.213037 | 1 | 0.714286 | 0 |
| 6717 | 14 | 0.037962 | 0.213037 | 1 | 0 | 0.714286 |
| 6834 | 14 | 0.037962 | 0.213037 | 1 | 0 | 1 |
| 7143 | 11 | 0.037962 | 0.213037 | 1 | 0.285714 | 1.285714 |
| 90 | 9 | 0.038961 | 0.213037 | 1 | 36 | 8.428571 |
| 160 | 9 | 0.038961 | 0.213037 | 1 | 21.14286 | 418.5714 |
| 694 | 17.5 | 0.038961 | 0.213037 | 1 | 0.285714 | 0 |
| 709 | 14 | 0.038961 | 0.213037 | 1 | 0.714286 | 0 |
| 845 | 14 | 0.038961 | 0.213037 | 1 | 0 | 1.285714 |
| 3570 | 14 | 0.038961 | 0.213037 | 1 | 0 | 0.714286 |
| 3670 | 14 | 0.038961 | 0.213037 | 1 | 0 | 1.428571 |
| 3908 | 9.5 | 0.038961 | 0.213037 | 1 | 4.285714 | 0.857143 |
| 4671 | 14 | 0.038961 | 0.213037 | 1 | 17.85714 | 0 |
| 4724 | 14 | 0.038961 | 0.213037 | 1 | 0.857143 | 0 |
| 4785 | 9.5 | 0.038961 | 0.213037 | 1 | 9.285714 | 3.857143 |
| 4903 | 10 | 0.038961 | 0.213037 | 1 | 1.142857 | 12.85714 |
| 5192 | 14 | 0.038961 | 0.213037 | 1 | 1.857143 | 0 |
| 5986 | 14 | 0.038961 | 0.213037 | 1 | 0 | 3.571429 |
| 6255 | 12.5 | 0.038961 | 0.213037 | 1 | 0.142857 | 2.142857 |
| 6329 | 14 | 0.038961 | 0.213037 | 1 | 0 | 1 |
| 6365 | 14 | 0.038961 | 0.213037 | 1 | 0 | 0.857143 |
| 6381 | 14 | 0.038961 | 0.213037 | 1 | 0 | 1.714286 |
| 6710 | 14 | 0.038961 | 0.213037 | 1 | 0 | 0.571429 |
| 6725 | 14 | 0.038961 | 0.213037 | 1 | 0 | 0.571429 |
| 8410 | 14 | 0.038961 | 0.213037 | 1 | 0 | 1.857143 |
| 215 | 14 | 0.03996 | 0.213037 | 1 | 0 | 0.714286 |
| 1357 | 17.5 | 0.03996 | 0.213037 | 1 | 0 | 0.571429 |
| 1613 | 14 | 0.03996 | 0.213037 | 1 | 0 | 0.571429 |
| 1674 | 9 | 0.03996 | 0.213037 | 1 | 9.142857 | 41 |
| 1793 | 14 | 0.03996 | 0.213037 | 1 | 1.857143 | 0 |
| 2916 | 10 | 0.03996 | 0.213037 | 1 | 0.714286 | 12.14286 |
| 3449 | 14 | 0.03996 | 0.213037 | 1 | 0 | 0.857143 |
| 3801 | 14 | 0.03996 | 0.213037 | 1 | 0.857143 | 0 |
| 4631 | 14 | 0.03996 | 0.213037 | 1 | 0.714286 | 0 |
| 4908 | 14 | 0.03996 | 0.213037 | 1 | 2.428571 | 0 |
| 5160 | 14 | 0.03996 | 0.213037 | 1 | 0.714286 | 0 |
| 6105 | 14 | 0.03996 | 0.213037 | 1 | 0 | 0.571429 |
| 7463 | 14 | 0.03996 | 0.213037 | 1 | 0 | 0.571429 |
| 8901 | 14 | 0.03996 | 0.213037 | 1 | 0 | 2.571429 |
| 122 | 10 | 0.040959 | 0.213037 | 1 | 116.2857 | 212.5714 |
| 125 | 10 | 0.040959 | 0.213037 | 1 | 2.714286 | 0.857143 |
| 157 | 9 | 0.040959 | 0.213037 | 1 | 124.7143 | 409.5714 |
| 158 | 9 | 0.040959 | 0.213037 | 1 | 1200.143 | 1895 |
| 2569 | 12 | 0.040959 | 0.213037 | 1 | 0.142857 | 2.285714 |
| 3139 | 17.5 | 0.040959 | 0.213037 | 1 | 0 | 0.285714 |
| 3158 | 14 | 0.040959 | 0.213037 | 1 | 5.857143 | 0 |
| 3945 | 17.5 | 0.040959 | 0.213037 | 1 | 0 | 0.285714 |
| 4182 | 14 | 0.040959 | 0.213037 | 1 | 0.714286 | 0 |
| 5336 | 14 | 0.040959 | 0.213037 | 1 | 2.142857 | 0 |
| 6026 | 14 | 0.040959 | 0.213037 | 1 | 0 | 1.142857 |
| 7095 | 17.5 | 0.040959 | 0.213037 | 1 | 0.285714 | 0 |
| 8594 | 14 | 0.040959 | 0.213037 | 1 | 0 | 0.714286 |
| 8832 | 17.5 | 0.040959 | 0.213037 | 1 | 0.285714 | 0 |
| 425 | 9 | 0.041958 | 0.213037 | 1 | 19.57143 | 41.71429 |
| 504 | 9.5 | 0.041958 | 0.213037 | 1 | 1.142857 | 6.428571 |
| 540 | 14 | 0.041958 | 0.213037 | 1 | 0 | 2 |
| 620 | 14 | 0.041958 | 0.213037 | 1 | 0 | 1.142857 |
| 1042 | 9 | 0.041958 | 0.213037 | 1 | 153.2857 | 757.4286 |
| 1302 | 14 | 0.041958 | 0.213037 | 1 | 2.142857 | 0 |
| 1560 | 10.5 | 0.041958 | 0.213037 | 1 | 3.142857 | 0.428571 |
| 1806 | 9 | 0.041958 | 0.213037 | 1 | 6.142857 | 1.714286 |
| 1904 | 11 | 0.041958 | 0.213037 | 1 | 1 | 3.142857 |
| 2019 | 14 | 0.041958 | 0.213037 | 1 | 0.142857 | 0.571429 |
| 2258 | 11 | 0.041958 | 0.213037 | 1 | 2.857143 | 0.285714 |
| 2408 | 14 | 0.041958 | 0.213037 | 1 | 0 | 2 |
| 2520 | 10 | 0.041958 | 0.213037 | 1 | 3.285714 | 0.857143 |
| 3002 | 14 | 0.041958 | 0.213037 | 1 | 0 | 1.285714 |
| 3294 | 12.5 | 0.041958 | 0.213037 | 1 | 0.142857 | 1.285714 |
| 3568 | 17.5 | 0.041958 | 0.213037 | 1 | 0 | 0.285714 |
| 4064 | 10 | 0.041958 | 0.213037 | 1 | 3.571429 | 0.285714 |
| 4114 | 12.5 | 0.041958 | 0.213037 | 1 | 0.142857 | 1 |
| 7496 | 14 | 0.041958 | 0.213037 | 1 | 1.857143 | 0 |
| 54 | 8.5 | 0.042957 | 0.213037 | 1 | 17.57143 | 2.428571 |
| 145 | 9.5 | 0.042957 | 0.213037 | 1 | 160 | 83.57143 |
| 340 | 9.5 | 0.042957 | 0.213037 | 1 | 104.8571 | 17.28571 |
| 624 | 10 | 0.042957 | 0.213037 | 1 | 0.428571 | 3.571429 |
| 647 | 12 | 0.042957 | 0.213037 | 1 | 4.714286 | 0.142857 |
| 981 | 11 | 0.042957 | 0.213037 | 1 | 1.571429 | 0.285714 |
| 1046 | 17.5 | 0.042957 | 0.213037 | 1 | 0.285714 | 0 |
| 1090 | 10 | 0.042957 | 0.213037 | 1 | 6.142857 | 0.285714 |
| 2342 | 9 | 0.042957 | 0.213037 | 1 | 818 | 360 |
| 2625 | 9 | 0.042957 | 0.213037 | 1 | 13.14286 | 2.142857 |
| 2704 | 10.5 | 0.042957 | 0.213037 | 1 | 0.428571 | 6 |
| 3551 | 10 | 0.042957 | 0.213037 | 1 | 3.428571 | 7.428571 |
| 3959 | 9.5 | 0.042957 | 0.213037 | 1 | 0.428571 | 2.285714 |
| 4396 | 14 | 0.042957 | 0.213037 | 1 | 0 | 2.857143 |
| 4625 | 14 | 0.042957 | 0.213037 | 1 | 1 | 0 |
| 5643 | 12.5 | 0.042957 | 0.213037 | 1 | 0.142857 | 1.714286 |
| 6001 | 17.5 | 0.042957 | 0.213037 | 1 | 0 | 0.571429 |
| 6092 | 17.5 | 0.042957 | 0.213037 | 1 | 0 | 0.285714 |
| 6283 | 17.5 | 0.042957 | 0.213037 | 1 | 0 | 0.285714 |
| 6639 | 14 | 0.042957 | 0.213037 | 1 | 0 | 1.714286 |
| 7464 | 14 | 0.042957 | 0.213037 | 1 | 0 | 2.142857 |
| 7652 | 17.5 | 0.042957 | 0.213037 | 1 | 0.285714 | 0 |
| 7989 | 14 | 0.042957 | 0.213037 | 1 | 0 | 0.571429 |
| 8737 | 17.5 | 0.042957 | 0.213037 | 1 | 0.285714 | 0 |
| 198 | 10 | 0.043956 | 0.213037 | 1 | 7 | 644 |
| 217 | 17.5 | 0.043956 | 0.213037 | 1 | 0.285714 | 0 |
| 746 | 14 | 0.043956 | 0.213037 | 1 | 1 | 0 |
| 1306 | 10 | 0.043956 | 0.213037 | 1 | 2.142857 | 0.285714 |
| 1341 | 9.5 | 0.043956 | 0.213037 | 1 | 8.714286 | 2.142857 |
| 2002 | 12.5 | 0.043956 | 0.213037 | 1 | 1 | 0.142857 |
| 2051 | 9.5 | 0.043956 | 0.213037 | 1 | 1.142857 | 8.571429 |
| 2090 | 14 | 0.043956 | 0.213037 | 1 | 3 | 0 |
| 2261 | 10 | 0.043956 | 0.213037 | 1 | 83.57143 | 83 |
| 3210 | 17.5 | 0.043956 | 0.213037 | 1 | 0 | 0.285714 |
| 3398 | 14 | 0.043956 | 0.213037 | 1 | 0 | 1.571429 |
| 3617 | 17.5 | 0.043956 | 0.213037 | 1 | 0.285714 | 0 |
| 3708 | 17.5 | 0.043956 | 0.213037 | 1 | 0 | 0.285714 |
| 4384 | 17.5 | 0.043956 | 0.213037 | 1 | 0 | 0.285714 |
| 4472 | 17.5 | 0.043956 | 0.213037 | 1 | 0.285714 | 0 |
| 5193 | 14 | 0.043956 | 0.213037 | 1 | 0.571429 | 0 |
| 6408 | 14 | 0.043956 | 0.213037 | 1 | 0 | 1.428571 |
| 7651 | 14 | 0.043956 | 0.213037 | 1 | 0.571429 | 0 |
| 7923 | 14 | 0.043956 | 0.213037 | 1 | 0 | 0.571429 |
| 8559 | 17.5 | 0.043956 | 0.213037 | 1 | 0.285714 | 0 |
| 1744 | 14 | 0.044955 | 0.213037 | 1 | 0.857143 | 0 |
| 2340 | 9 | 0.044955 | 0.213037 | 1 | 11.71429 | 3.142857 |
| 3066 | 17.5 | 0.044955 | 0.213037 | 1 | 0 | 0.285714 |
| 3613 | 12 | 0.044955 | 0.213037 | 1 | 2 | 0.142857 |
| 3653 | 14 | 0.044955 | 0.213037 | 1 | 0.142857 | 0.571429 |
| 4411 | 12 | 0.044955 | 0.213037 | 1 | 3.428571 | 0.142857 |
| 4844 | 12 | 0.044955 | 0.213037 | 1 | 0.285714 | 1.428571 |
| 5478 | 17.5 | 0.044955 | 0.213037 | 1 | 0.285714 | 0 |
| 6574 | 17.5 | 0.044955 | 0.213037 | 1 | 0 | 0.285714 |
| 6580 | 17.5 | 0.044955 | 0.213037 | 1 | 0 | 0.285714 |
| 6900 | 17.5 | 0.044955 | 0.213037 | 1 | 0 | 0.285714 |
| 7100 | 17.5 | 0.044955 | 0.213037 | 1 | 0 | 0.428571 |
| 7610 | 11 | 0.044955 | 0.213037 | 1 | 0.285714 | 2.714286 |
| 286 | 17.5 | 0.045954 | 0.213037 | 1 | 0.285714 | 0 |
| 292 | 10 | 0.045954 | 0.213037 | 1 | 2.428571 | 0.285714 |
| 451 | 10 | 0.045954 | 0.213037 | 1 | 15.42857 | 74.57143 |
| 1190 | 14 | 0.045954 | 0.213037 | 1 | 0.714286 | 0 |
| 1271 | 9.5 | 0.045954 | 0.213037 | 1 | 4.285714 | 16 |
| 1925 | 12 | 0.045954 | 0.213037 | 1 | 2.428571 | 0.142857 |
| 2069 | 9.5 | 0.045954 | 0.213037 | 1 | 0.857143 | 3 |
| 2872 | 17.5 | 0.045954 | 0.213037 | 1 | 0.285714 | 0 |
| 3479 | 14 | 0.045954 | 0.213037 | 1 | 0 | 1.428571 |
| 3728 | 17.5 | 0.045954 | 0.213037 | 1 | 0.285714 | 0 |
| 4157 | 17.5 | 0.045954 | 0.213037 | 1 | 0.285714 | 0 |
| 4681 | 17.5 | 0.045954 | 0.213037 | 1 | 0.285714 | 0 |
| 6018 | 17.5 | 0.045954 | 0.213037 | 1 | 0 | 0.285714 |
| 6236 | 10.5 | 0.045954 | 0.213037 | 1 | 0.714286 | 9.142857 |
| 6466 | 17.5 | 0.045954 | 0.213037 | 1 | 0.285714 | 0 |
| 6524 | 14 | 0.045954 | 0.213037 | 1 | 0.142857 | 0.571429 |
| 6595 | 12 | 0.045954 | 0.213037 | 1 | 0.142857 | 4 |
| 6638 | 17.5 | 0.045954 | 0.213037 | 1 | 0 | 0.285714 |
| 7601 | 17.5 | 0.045954 | 0.213037 | 1 | 0 | 0.285714 |
| 7997 | 17.5 | 0.045954 | 0.213037 | 1 | 0 | 0.285714 |
| 8626 | 17.5 | 0.045954 | 0.213037 | 1 | 0.285714 | 0 |
| 8943 | 17.5 | 0.045954 | 0.213037 | 1 | 0 | 0.285714 |
| 45 | 17.5 | 0.046953 | 0.213037 | 1 | 0.285714 | 0 |
| 128 | 11 | 0.046953 | 0.213037 | 1 | 6.714286 | 0.285714 |
| 335 | 10.5 | 0.046953 | 0.213037 | 1 | 3 | 0.428571 |
| 1548 | 12 | 0.046953 | 0.213037 | 1 | 2.857143 | 0.142857 |
| 1860 | 10 | 0.046953 | 0.213037 | 1 | 1.142857 | 4 |
| 1933 | 17.5 | 0.046953 | 0.213037 | 1 | 0 | 0.285714 |
| 2088 | 11 | 0.046953 | 0.213037 | 1 | 0.714286 | 5.857143 |
| 2343 | 9 | 0.046953 | 0.213037 | 1 | 44.28571 | 294.8571 |
| 2362 | 12.5 | 0.046953 | 0.213037 | 1 | 0.142857 | 1.285714 |
| 2529 | 11 | 0.046953 | 0.213037 | 1 | 0.285714 | 3.285714 |
| 2973 | 14 | 0.046953 | 0.213037 | 1 | 0 | 0.857143 |
| 3413 | 10 | 0.046953 | 0.213037 | 1 | 1 | 4.428571 |
| 3667 | 17.5 | 0.046953 | 0.213037 | 1 | 0.285714 | 0 |
| 4643 | 17.5 | 0.046953 | 0.213037 | 1 | 0 | 0.285714 |
| 5075 | 14 | 0.046953 | 0.213037 | 1 | 1.571429 | 0 |
| 5124 | 17.5 | 0.046953 | 0.213037 | 1 | 0.857143 | 0 |
| 5611 | 17.5 | 0.046953 | 0.213037 | 1 | 0.285714 | 0 |
| 5809 | 10 | 0.046953 | 0.213037 | 1 | 2.571429 | 6.571429 |
| 6410 | 17.5 | 0.046953 | 0.213037 | 1 | 0 | 0.285714 |
| 6579 | 14 | 0.046953 | 0.213037 | 1 | 0 | 0.571429 |
| 7421 | 17.5 | 0.046953 | 0.213037 | 1 | 0.285714 | 0 |
| 7927 | 17.5 | 0.046953 | 0.213037 | 1 | 0 | 0.285714 |
| 8213 | 17.5 | 0.046953 | 0.213037 | 1 | 0.285714 | 0 |
| 182 | 9 | 0.047952 | 0.213037 | 1 | 10.71429 | 42 |
| 1410 | 11 | 0.047952 | 0.213037 | 1 | 1 | 39.85714 |
| 2282 | 12.5 | 0.047952 | 0.213037 | 1 | 0.142857 | 1 |
| 3144 | 17.5 | 0.047952 | 0.213037 | 1 | 0.285714 | 0 |
| 3677 | 14 | 0.047952 | 0.213037 | 1 | 1.142857 | 0 |
| 4192 | 17.5 | 0.047952 | 0.213037 | 1 | 0 | 0.285714 |
| 5111 | 17.5 | 0.047952 | 0.213037 | 1 | 0.285714 | 0 |
| 5167 | 17.5 | 0.047952 | 0.213037 | 1 | 0.285714 | 0 |
| 5332 | 13 | 0.047952 | 0.213037 | 1 | 0.857143 | 0.142857 |
| 5902 | 17.5 | 0.047952 | 0.213037 | 1 | 0.285714 | 0 |
| 5977 | 14 | 0.047952 | 0.213037 | 1 | 0 | 0.571429 |
| 6002 | 17.5 | 0.047952 | 0.213037 | 1 | 0.285714 | 0 |
| 6262 | 17.5 | 0.047952 | 0.213037 | 1 | 0 | 0.285714 |
| 6832 | 12 | 0.047952 | 0.213037 | 1 | 0.142857 | 2.857143 |
| 6890 | 17.5 | 0.047952 | 0.213037 | 1 | 0 | 0.285714 |
| 8172 | 10.5 | 0.047952 | 0.213037 | 1 | 0.714286 | 2.714286 |
| 8506 | 17.5 | 0.047952 | 0.213037 | 1 | 0 | 0.285714 |
| 311 | 17.5 | 0.048951 | 0.213037 | 1 | 0 | 0.285714 |
| 395 | 10 | 0.048951 | 0.213037 | 1 | 20.85714 | 88.57143 |
| 1351 | 17.5 | 0.048951 | 0.213037 | 1 | 0.285714 | 0 |
| 1382 | 17.5 | 0.048951 | 0.213037 | 1 | 0.285714 | 0 |
| 1932 | 9.5 | 0.048951 | 0.213037 | 1 | 12 | 5 |
| 2213 | 9.5 | 0.048951 | 0.213037 | 1 | 2.428571 | 18 |
| 2253 | 10 | 0.048951 | 0.213037 | 1 | 0.428571 | 19.71429 |
| 2344 | 17.5 | 0.048951 | 0.213037 | 1 | 0.857143 | 0 |
| 2981 | 10.5 | 0.048951 | 0.213037 | 1 | 1.142857 | 3.857143 |
| 3849 | 17.5 | 0.048951 | 0.213037 | 1 | 0.285714 | 0 |
| 5178 | 9.5 | 0.048951 | 0.213037 | 1 | 5.857143 | 2.571429 |
| 5467 | 17.5 | 0.048951 | 0.213037 | 1 | 0.285714 | 0 |
| 6150 | 17.5 | 0.048951 | 0.213037 | 1 | 0 | 0.285714 |
| 6276 | 17.5 | 0.048951 | 0.213037 | 1 | 0 | 0.285714 |
| 6393 | 17.5 | 0.048951 | 0.213037 | 1 | 0 | 0.285714 |
| 6789 | 17.5 | 0.048951 | 0.213037 | 1 | 0 | 0.285714 |
| 7187 | 17.5 | 0.048951 | 0.213037 | 1 | 0 | 0.285714 |
| 7595 | 11 | 0.048951 | 0.213037 | 1 | 1.285714 | 0.285714 |
| 9025 | 17.5 | 0.048951 | 0.213037 | 1 | 0.285714 | 0 |
| 708 | 17.5 | 0.04995 | 0.213037 | 1 | 0 | 0.285714 |
| 726 | 11 | 0.04995 | 0.213037 | 1 | 0.428571 | 1.142857 |
| 753 | 9.5 | 0.04995 | 0.213037 | 1 | 13.14286 | 5.571429 |
| 862 | 11 | 0.04995 | 0.213037 | 1 | 0.285714 | 2.142857 |
| 1321 | 12 | 0.04995 | 0.213037 | 1 | 0.142857 | 4.857143 |
| 1412 | 17.5 | 0.04995 | 0.213037 | 1 | 0.285714 | 0 |
| 1545 | 17.5 | 0.04995 | 0.213037 | 1 | 0.285714 | 0 |
| 1824 | 10 | 0.04995 | 0.213037 | 1 | 3.714286 | 1.428571 |
| 2980 | 17.5 | 0.04995 | 0.213037 | 1 | 0.285714 | 0 |
| 3213 | 11 | 0.04995 | 0.213037 | 1 | 0.428571 | 1.857143 |
| 3464 | 9.5 | 0.04995 | 0.213037 | 1 | 32.14286 | 1.571429 |
| 3465 | 12 | 0.04995 | 0.213037 | 1 | 0.142857 | 2.428571 |
| 3486 | 17.5 | 0.04995 | 0.213037 | 1 | 0 | 0.285714 |
| 3719 | 17.5 | 0.04995 | 0.213037 | 1 | 0.285714 | 0 |
| 3918 | 12 | 0.04995 | 0.213037 | 1 | 0.142857 | 1.857143 |
| 3991 | 12 | 0.04995 | 0.213037 | 1 | 1.714286 | 0.142857 |
| 4167 | 10 | 0.04995 | 0.213037 | 1 | 8.571429 | 2.285714 |
| 4377 | 17.5 | 0.04995 | 0.213037 | 1 | 0 | 0.285714 |
| 4481 | 17.5 | 0.04995 | 0.213037 | 1 | 0.571429 | 0 |
| 4900 | 14 | 0.04995 | 0.213037 | 1 | 0.571429 | 0 |
| 5026 | 10.5 | 0.04995 | 0.213037 | 1 | 1.142857 | 5.428571 |
| 5337 | 17.5 | 0.04995 | 0.213037 | 1 | 0.285714 | 0 |
| 6252 | 17.5 | 0.04995 | 0.213037 | 1 | 0 | 0.285714 |
| 6377 | 11 | 0.04995 | 0.213037 | 1 | 0.571429 | 2.142857 |
| 7157 | 17.5 | 0.04995 | 0.213037 | 1 | 0 | 0.285714 |
| 7935 | 17.5 | 0.04995 | 0.213037 | 1 | 0.571429 | 0 |
| 9042 | 17.5 | 0.04995 | 0.213037 | 1 | 0 | 0.285714 |
