# Supplementary material for: Characterization of the gut microbiota and fecal metabolome in the osteosarcoma mouse model
Source: Aging (Albany NY). 2024 Jul 3;16(13):10841–59. doi: 10.18632/aging.205951 (PMC11272122; doi:10.18632/aging.205951)
Supplement: Supplementary Table 2 [file aging-16-205951-s003.pdf]

SUPPLEMENTARY TABLE

Supplementary Table 2. Metabolic pathways associated with bone metabolism in Top10 of KEGG.

ID	Annotation	p-value	-lg(p-value)	Matching
mmu00970	Aminoacyl-tRNA biosynthesis	1.09019681319946E-07	6.96249509181108	L-Lysine, L-Arginine, L-Glutamine, L-Serine, L-tryptophan, L-Phenylalanine, Benzenepropanoic acid, Leucine, L-Histidine, L-Proline, L-Valine, L-threonine, L-isoleucine
mmu04150	mTOR signaling pathway	0.000279653418412148	3.55337986759817	Adenosine-5'-monophosphate, L-Arginine, Leucine
mmu00330	Arginine and proline metabolism	0.00032755871560812	3.48471084046909	L-Arginine, Ornithine, Putrescine, L-proline, Creatine, Spermidine, 5-aminovaleric acid, Creatinine, 4-hydroxyproline, N2-Succinyl-L-ornithine, (2E)-N-(4-aminobutyl)-3-(4-hydroxy-3-methoxyphenyl)prop-2-enimidic acid
